# Exploring the dynamics and interactions of the N-myc transactivation domain through solution nuclear magnetic resonance spectroscopy

**DOI:** 10.1042/BCJ20240248

**Published:** 2024-10-21

**Authors:** Ewa Rejnowicz, Matthew Batchelor, Eoin Leen, Mohd Syed Ahangar, Selena G. Burgess, Mark W. Richards, Arnout P. Kalverda, Richard Bayliss

**Affiliations:** Astbury Centre for Structural Molecular Biology, School of Molecular and Cellular Biology, Faculty of Biological Sciences, University of Leeds, Leeds LS2 9JT, U.K.

**Keywords:** intrinsically disordered proteins, myc, neuroblastoma, NMR spectroscopy, phosphorylation/dephosphorylation, protein–protein interactions

## Abstract

Myc proteins are transcription factors crucial for cell proliferation. They have a C-terminal domain that mediates Max and DNA binding, and an N-terminal disordered region culminating in the transactivation domain (TAD). The TAD participates in many protein–protein interactions, notably with kinases that promote stability (Aurora-A) or degradation (ERK1, GSK3) via the ubiquitin-proteasome system. We probed the structure, dynamics and interactions of N-myc TAD using nuclear magnetic resonance (NMR) spectroscopy following its complete backbone assignment. Chemical shift analysis revealed that N-myc has two regions with clear helical propensity: Trp77–Glu86 and Ala122–Glu132. These regions also have more restricted ps–ns motions than the rest of the TAD, and, along with the phosphodegron, have comparatively high transverse (*R*_2_) ^15^N relaxation rates, indicative of slower timescale dynamics and/or chemical exchange. Collectively these features suggest differential propensities for structure and interaction, either internal or with binding partners, across the TAD. Solution studies on the interaction between N-myc and Aurora-A revealed a previously uncharacterised binding site. The specificity and kinetics of sequential phosphorylation of N-myc by ERK1 and GSK3 were characterised using NMR and resulted in no significant structural changes outside the phosphodegron. When the phosphodegron was doubly phosphorylated, N-myc formed a robust interaction with the Fbxw7–Skp1 complex, but mapping the interaction by NMR suggests a more extensive interface. Our study provides foundational insights into N-myc TAD dynamics and a backbone assignment that will underpin future work on the structure, dynamics, interactions and regulatory post-translational modifications of this key oncoprotein.

## Introduction

N-myc belongs to a family of transcription factor proteins which includes two other members, L-myc and c-myc. All three members are expressed in mammals and have functions in growth and development, but also have well documented pathological roles as oncoproteins in a wide variety of cancers [1–3]. The N-myc gene was discovered in a neuroblastoma cell line as a copy number amplified DNA sequence with sequence similarity to c-myc [4]. It is an important driver for cancers of neuronal origin such as neuroblastoma, medulloblastoma and retinoblastoma [5]. Consistent with this role as a driver oncogene, copy number amplification of N-myc is correlated with poor clinical outcomes in neuroblastoma [6–8]. The more extensively studied c-myc has been shown to act as a global amplifier of transcription, acting to increase transcription of already active genes [9–11]. c-myc activates transcription of Pol I (ribosomal RNA), Pol II (mRNA), and Pol III (short, structured RNA) genes [9,11–15]. However, at specific loci c-myc has also been shown to act as a transcriptional repressor, at least in relative terms [16,17].

Myc proteins vary in size from 364 residues for L-myc to 464 residues for N-myc but have similar domain structures. The C-terminus of the protein contains a basic helix-loop-helix leucine zipper sequence of ∼90 residues, which forms a hetero-dimer with Max to produce a DNA binding domain that *bind* E-box sequences in the promoters and enhancers of genes [18–23]. The N-terminal region of the protein (∼370 residues in N-myc) is thought to be intrinsically disordered. This intrinsic disorder means that many of the functions of myc are brought about through recruitment of enzymes or structural proteins to sites close to E-box DNA sequences. Consistent with this role, mass-spec proteomics experiments have demonstrated that myc binds to hundreds of other proteins, either directly, or indirectly [24,25]. There are six regions of conservation, known as myc boxes (MB), in the N-terminal region of the protein. Transgenic mice with N-myc replacing c-myc are both viable and reproduction competent [26]. This functional redundancy implies that the conserved sequences can mediate many, if not all, of the essential roles of myc. The transactivation domain (TAD) of myc transcription factors was defined in c-myc (residues 1–143) and contains the first three of these myc boxes (MB0, MBI, and MBII) [27]. Deletion of the first 150 residues of c-myc has been shown to abrogate both the capacity of c-myc to act as a transcriptional activator and to transform Rat-1a cells [28]. Recently, N-myc at elevated concentrations in neuroblastoma cells was shown to enter phase separated condensates in the nucleus, and that these condensates could differentially regulate gene expression. The TAD of N-myc (residues 1–137) was essential for this phase separation, further highlighting the importance of this part of the protein in myc-mediated oncogenesis [29].

Some of the specific functions of myc boxes and other sequences within the TAD have been identified. MB0 (N-myc 18–37) and MBII (N-myc 110–126) recruit proteins such as histone acetyltransferase complexes (e.g. STAGA, TIP60), polymerase associated factors (e.g. general transcription factor TFIIF), and other known transcriptional regulators (e.g. transcriptional intermediary factor 1α) [25]. In both c-myc and N-myc, the poorly conserved sequence between MBI (N-myc 45–63) and MBII is known to interact with molecules important for myc function. In c-myc, this region interacts with the general transcription factor complex, TFIID, part of the Polymerase II Pre-initiation complex [30]. In N-myc, this region interacts with Aurora-A kinase, which is an important complex for prevention of transcription–replication conflicts in S-phase [31,32]. Recently a novel function of c-myc MB0 has been determined using nuclear magnetic resonance (NMR). MB0 binds to the DNA binding domain of the c-myc–Max dimer and appears to play a regulatory role in the complex binding to DNA [33]. MBI acts as a phosphodegron and is highly conserved across myc proteins; N- and c-myc have identical core MBI sequences (^47^EDIWKKFELLPTPPLSP^63^). Myc is phosphorylated at Ser62 either by ERK kinases, downstream of growth signalling, or by cell cycle kinases such as CDK1 or CDK2 [34–36]. Phosphorylation of Ser62 primes phosphorylation at Thr58 by GSK3 [34]. Thr58–Ser62 doubly phosphorylated species can bind to the Fbxw7 protein which is part of the of SCF^Fbxw7^ E3 ubiquitin ligase complex, this interaction results in ubiquitination of myc and subsequent proteasomal degradation [37–41]. In c-myc the MBI phosphodegron acts in concert with another phosphodegron around residue 244. An equivalent second phosphodegron has yet to be found for N-myc or L-myc [37]. This system is responsible for the short half-life of myc in cells, which for c-myc is 20–30 min [42].

Myc proteins are, for the most part, intrinsically disordered proteins (IDPs), they are regulated by post-translational modifications, interact with a large number of proteins, and tend to interact with these binding partners in the low micromolar range (e.g. Aurora-A 1 μM; GTFIIF 4.9 μM; TBP-TAF1 5.2 μM; Bin1 4.2 μM; PNUTS 3.5 μM) [25,30,31,43,44]. For these reasons NMR spectroscopy is perhaps the most appropriate method to understand their structure, dynamics, regulation, and interactions. To a significant extent, previous NMR studies on myc have focused on the basic helix-loop-helix DNA binding domain and its interaction with Max [45–52]. The majority of backbone resonances for part of the c-myc TAD [1–88], which includes MB0 and MBI, have also been assigned [44]. This has been extremely useful to understand the dynamics, transient secondary structure, and effects of phosphorylation in this sequence. It has also been used to characterise the interaction between myc and binding partners such as PIN1 and Bin1 [44,53]. Recently, backbone assignments have been determined for a large proportion of the c-myc backbone outside of the TAD and dimerisation domain. The assignment was achieved using a divide and conquer approach using two polypeptides, c-myc 151–255 and 256–351 [33]. However, to date NMR has not been used to investigate the TAD of N-myc.

Here we present the first backbone NMR assignment of the N-myc TAD. This includes the first myc assignment for MBII and the region known to interact with Aurora-A as a helix [31]. We characterise the dynamics of the molecule across timescales and orthogonally validate the presence of elements with latent secondary structure using circular dichroism (CD) spectroscopy. Additionally, we use NMR titrations to help explore the N-myc–Aurora-A complex, incorporating/implicating MBII as a further Aurora-A-binding region within an extensive interaction profile between the two proteins. Finally, we use the assignment to characterise the effects on N-myc structure of the MBI phosphorylation series and demonstrate phosphorylation-dependent and phosphorylation-independent complex formation with Fbxw7–Skp1.

## Experimental procedures

### Expression constructs

N-myc_1–137_ TAD and N-myc_64–137_ constructs were cloned into a pETM6T1 vector which contains a TEV-cleavable N-terminal His-NusA solubility/expression tag as described previously [54]. The construct for N-terminally 3xFLAG-tagged N-myc_1–137_ was generated in previous work [31]. N-myc_18–59_ and N-myc_18–72_ sequences with a C27S mutation were cloned into a pETM6T1 vector which contains the same TEV-cleavable N-terminal His-NusA tag followed by GB1 [55]. TEV cleavage leaves a short sequence GAM or GAMG in the constructs prior to Met1 in N-myc TAD, or Ser64 in N-myc_64–137_, respectively. N-myc_1–137_ TAD S7A was produced by site-directed mutagenesis using the Stratagene QuikChange protocol. TEV cleavage leaves a 62-residue sequence, including that of GB1, prior to Asp18 in GB1-N-myc proteins. The gene sequence of Fbxw7_261–706_ was cloned into a pET30-TEV vector with a TEV-cleavable 6x-His tag and the gene sequence of Skp1 was cloned into a pCDF-duet vector with no affinity tag. The Aurora-A kinase domain 122–403 C290A,C393A construct used was previously described [56].

### Protein production

Proteins were expressed using BL21 (DE3) RIL *Escherichia coli* cells. Cells were transformed using the heat-shock method with the vector(s) of interest. 35 μg/ml chloramphenicol was used for routine maintenance of the RIL vector. 50 μg/ml kanamycin and 100 μg/ml spectinomycin was used to select and maintain pET and pCDF vectors, respectively. A single colony was grown overnight in LB at 200 rpm and 37°C. Ten millilitres of this starter culture was used to seed each litre of LB media. The cells were grown to mid-log phase. For routine expression, cells were then induced by addition of 0.6 mM ITPG and incubated overnight at 200 rpm and 20°C. Cells were harvested by centrifugation at ∼6000 ***g*** for 20 min. Pellets were either stored at −80°C or processed immediately. For NMR-labelled proteins, LB cultures were grown to mid-log phase in LB as described previously, but instead of being induced they were first pelleted at 2500 ***g*** at 20°C for 20 min. Cell pellets were resuspended in PBS buffer to remove residual LB. Bacterial cultures were then centrifuged again as described above. The resultant pellets were resuspended in 250 ml of minimal media (for each litre of LB), transferred to an autoclaved 2.5 l flask and incubated at 20°C for a further 2 h at 200 rpm. Following this incubation period, overnight expression was induced with 0.6 mM IPTG. Cells were finally harvested as outlined previously. Minimal media consisted of 0.002 g/ml of ^15^NH_4_Cl and 0.01 g/ml of ^13^C glucose (or unlabelled glucose for ^15^N-only expression) in 50 mM Na_2_HPO_4_, 25 mM KH_2_PO_4_, 20 mM NaCl supplemented with 2 mM MgSO_4_, 0.2 mM CaCl_2_, 0.01 mM FeSO_4_.7H_2_O, micronutrients and a vitamin solution (BME vitamins 100× solution, Sigma–Aldrich). The solution was syringe filtered (0.2 μm) prior to use. Labelled 3xFLAG-N-myc_1–137_ was produced as described previously using ^15^NH_4_Cl supplemented M9 media [57].

### Protein purification

To purify N-myc_1–137_ and N-myc_64–137_, bacterial pellets were resuspended using TBS-TCEP buffer (25 mM Tris, 2.7 mM KCl, 137 mM NaCl, 2 mM tris(2-carboxyethyl)phosphine (TCEP), pH 6.9) supplemented with c0mplete™ Mini EDTA-free Protease Inhibitor Cocktail tablets (Roche) and 10 mg lysozyme. Cells were lysed by sonication and then clarified by centrifugation at 50 000 ***g*** for 1 h. Protein was initially purified using affinity chromatography using HIS-Select Cobalt Affinity Gel (Sigma) on a gravity flow column. TEV protease was added, and the solution was dialysed in TBS-TCEP buffer at 4°C overnight. Dialysed protein was further purified by ion-exchange chromatography using a HiTrap-FF Q column (GE Healthcare), resulting in the elution of N-myc proteins in the flow-through and at low salt concentrations. Fractions containing N-myc were subjected to a Cobalt affinity subtraction step to remove His-tagged TEV protease. Finally, N-myc proteins were polished using size-exclusion chromatography (SEC) using a Superdex 16/600 S75 column (GE Healthcare). GB1-tagged N-myc proteins were purified in a similar manner with a few alterations: 25 mM MES, 200 mM NaCl, 5 mM β-mercaptoethanol, pH 6.5 was used instead of the TBS–TCEP buffer. The dialysis buffer was the same with the exception that the NaCl concentration was 150 mM. Ion exchange was not performed on these proteins. The final size exclusion buffer contained 20 mM (K/H)_3_PO_4_, 150 mM NaCl, pH 6.5. 3xFLAG-N-myc_1–137_ was purified as described in previous work [31], with final SEC step into TBS-TCEP buffer.

Fbxw7 and Skp1 were purified using Ni-affinity chromatography followed by dialysis with TEV protease to cleave the His-tag on Fbxw7. The cleaved tag was removed by applying the dialysed protein again to the Ni-column. Fractions containing purified complex were concentrated and subjected to SEC using a Superdex 16/600 S200 column equilibrated in 20 mM Tris, 200 mM NaCl, 2 mM β-mercaptoethanol, 10% glycerol, pH 8.

The kinase domain of Aurora-A (Aurora-A 122–403, C290A, C393A) was purified as described previously [56]. Protein concentrations were measured by absorbance at 280 nm using extinction coefficients determined using the Expasy ProtParam tool (https://web.expasy.org/cgi-bin/protparam/protparam). Purified proteins were concentrated, aliquoted and used immediately or snap frozen in liquid nitrogen and stored at −80°C prior to use.

### NMR spectroscopy

The majority of NMR spectra were recorded on a 750-MHz Oxford Instruments magnet equipped with a Bruker Avance console and TCI cryoprobe. Additional experiments were recorded on a 950-MHz Bruker Ascend Aeon spectrometer equipped with a TXO cryoprobe and a 600-MHz Oxford Instruments magnet equipped with a Bruker Avance console and a QCI-P-cryoprobe (a list of experiments is given in Supplementary Table S1). ^1^H–^15^N HSQC spectra were recorded between 10 and 37°C, and peak positions tracked to transfer assignments across temperatures. Although variable across the sequence, intensities for the majority of peaks dropped at higher temperatures (Supplementary Figure S1B), so, coupled with a desire to prolong the lifetime of the sample, assignment spectra were recorded at 10°C. The data were processed into spectra using NMRPipe/NMRDraw [58]; peak assignments and further analysis were carried out with CCPNmr Analysis (version 2.5) [59]. For calculations of secondary shifts (Δδ), reference coil values generated using a web server hosted by the University of Copenhagen (https://www1.bio.ku.dk/english/research/bms/sbinlab/randomchemicalshifts2/ [60]) were subtracted from measured shift values. Further structural analysis using all measured shifts was carried out using the TALOS-N web server (https://spin.niddk.nih.gov/bax-apps/nmrserver/talosn/ [61]). Chemical shift perturbations (CSPs) were calculated using a 0.15 weighting for ^15^N shifts compared with ^1^H shifts.

^15^N longitudinal (*R*_1_) and transverse (*R*_2_) relaxation rates and ^15^N–^1^H heteronuclear NOEs were measured for resolved ^1^H–^15^N peaks of a 150 µM sample of N-myc TAD using the 600 MHz instrument. Spectra were recorded at 10°C. The recycle delays were 2.5 s for *R*_1_ and *R*_2_ experiments and 5 s for the heteronuclear NOE experiment. Eleven relaxation periods ranging from 10 to 1600 ms were used for the *R*_1_ experiment, and 12 relaxation periods from 17 to 201 ms were used for the *R*_2_ experiment. In both cases, two of the relaxation periods were duplicated to facilitate error estimations. Peak intensities measurements and analysis of relaxation rates was performed using PINT [62,63]. Some residues were excluded from these analyses due to peak overlap or where it was not possible to confidently follow peak intensity changes across the experiment.

Phosphorylation of ^15^N-labelled N-myc TAD was tracked by collection of ^1^H–^15^N HSQC spectra prior to and subsequent to addition of kinases [64]. TBS–TCEP buffer was supplemented with 4 mM MgCl_2_ and 1 mM ATP, and a concentration of 120 μM N-myc TAD was used. ERK1 and GSK3 kinases were purchased from the MRC Protein Phosphorylation Unit, University of Dundee, U.K. Small aliquots (∼10 μl) were added step-wise to give a final kinase concentration of ∼300 nM. By way of control, there was no sign of phosphorylation of N-myc TAD by Aurora-A or by PLK1 kinases. Direct phosphorylation with GSK3 was tested using ^15^N-labelled 3xFLAG-N-myc_1–137_. Peak assignment was unchanged and transferrable from untagged N-myc TAD for residues Cys4–Gly137. Barring a single FLAG Gly residue, peaks for the FLAG tag and N-myc 1–3 residues are not specifically assigned but changes in their peak intensities are reported as a block.

For NMR interaction studies, N-myc constructs and Aurora-A kinase domain (122–403, C290A, C393A) were separately buffer exchanged into the following buffer (20 mM (K/H)_3_PO_4_ pH 6.5, 150 mM NaCl, 2 mM β-mercaptoethanol, 1% glycerol, 5 mM MgCl_2_, 5 mM ADP). Buffer exchange was achieved by sequential dilution and concentration in centrifugal concentrators. Initial N-myc protein concentrations were 150–315 μM. ^1^H–^15^N HSQC peak positions were very similar to those in the original buffer so assignments were straightforwardly copied across. Aliquots of Aurora-A (305–400 μM were added up to a [N-myc]:[Aurora-A] molar ratio of 1:1.4.

For Fbxw7–Skp1 interaction studies, ∼50 μM samples of ^15^N-labelled 3xFLAG-N-myc_1–137_ were used. After an initial ^1^H–^15^N HSQC spectrum was recorded, doubly phosphorylated N-myc TAD was generated by treating the sample with a mixture of ERK1 (0.6 µM), GSK3 (0.3 µM), ATP (1 mM) and MgCl_2_ (5 mM) in TBS buffer. To generate singly-phosphorylated (pSer62) N-myc TAD, the GSK3 was omitted. After immediately recording a ^1^H–^15^N HSQC spectrum to confirm phosphorylation of Ser62 (and Thr58) had occurred, further off-target phosphorylation was quenched by addition of EDTA (final concentration 8 mM) to sequester Mg^2+^ ions. Quenching was confirmed by repeated ^1^H–^15^N HSQC spectra. Aliquots of Fbxw7–Skp1 (550 μM) were added up to a [N-myc]:[Fbxw7–Skp1] molar ratio of 1:2. An equivalent Fbxw7–Skp1 titration was carried out with unphosphorylated 3xFLAG-N-myc_1–137_. Throughout, error estimates (Δ*R*) for intensity ratios (*R*) were calculated using the NMRpipe command ‘showApod’ to measure the baseline noise in each spectrum (Δ*x*, Δ*y*). Errors were propagated using (Δ*R*/*R*)^2^ = (Δ*x*/*x*)^2^ + (Δ*y*/*y*)^2^, where *x* and *y* are peak intensities in each spectrum.

### CD spectroscopy of N-myc peptides

The following 17-residue N- and C-capped peptides were purchased from Biomatik Corp., Canada: Ac-PGEDIWKKFELLPTPPL-NH_2_ (N-myc 45–61, MBI); Ac-EPPSWVTEMLLENELWG-NH_2_ (N-myc 73–89, AIH); Ac-GFSAREKLERAVSEKLQ-NH_2_ (N-myc 119–135, MBII+); Ac-GFSAAAKLVSEKLASYQ-NH_2_ (c-myc 137–153, MBII+). Peptides were initially dissolved to give 0.5–1.0 mM stock solutions in buffer composed of 1 mM sodium borate, 1 mM sodium citrate, 1 mM (Na/H)_3_PO_4_ and 10 mM NaCl, pH 7 [65,66]. For measurements, samples were further diluted in buffer to concentrations of 10–100 µM. Equivalent concentration solutions were also prepared containing 15 or 30% (v/v) trifluoroethanol. Spectra were recorded using ∼200 μl samples in 1 mm pathlength quartz cuvettes and an APP Chirascan CD spectropolarimeter. To maximise the secondary structural content, spectra were recorded at 5°C. Data were collected every 1 nm for wavelengths 260–180 nm (1 nm s^−1^); each spectrum was recorded in duplicate. Due to significant Cl^–^ absorption, measurements <190 nm were unreliable and are not included. Background spectra were recorded using buffer alone, buffer/15% TFE or buffer/30% TFE, and background ellipticity values were subtracted from raw sample ellipticity values (*θ*(*λ*)) to calculate MREs using:

$$\text{MRE}(\lambda )=\frac{(\theta (\lambda )-\theta_{\;\mathrm{buffer}}(\lambda ))\times M_{w}}{\left(n\times \mathrm{concentration}\;\mathrm{in}\;\mathrm{mg}/\mathrm{ml}\right)}$$

where *M*_w_ is the peptide molecular mass, and *n* is the number of total peptide bonds, in each case taken to be 17 accounting for the acetyl capping group. Helicity values were estimated using the following equation:

$$\text{\%}\;\mathrm{helix}=(\mathrm{MR}E_{222}\text{–}415)/350$$

with MRE_222_ in units of deg cm^2^ dmol^−1^ res^−1^ [65,67].

### Analytical SEC

Analytical SEC was used to investigate the binding of N-myc TAD to the Fbxw7–Skp1 complex. Sample phosphorylation was achieved by addition of 1 mM ATP, 4 mM MgCl_2_, and 0.3 µM ERK1 and/or GSK3, and followed by NMR, and then samples were flash frozen to prevent further reaction. Prior to analytical SEC, N-myc TAD, in different phosphorylation states, was mixed in equivalent molar ratio with recombinant Fbxw7–Skp1 to a total volume of 100 µl. The proteins were incubated by slow rotation at 4°C for 2 h prior to SEC. SEC was performed using a ÄKTA pure FPLC system and a Superose 12 10/300 GL column. The loop and sample volumes were 60 and 100 μl respectively. The flow rate was 0.5 ml/min. Fractions were collected every 0.5 ml and analysed using SDS–PAGE.

### Coprecipitation assay

Peptide coprecipitation assays with Aurora-A kinase domain were carried out as described previously [31]. Peptides were synthesized by Peptide Protein Research Ltd, U.K.

## Results

### Backbone assignment of N-myc TAD

**Figure 1.**
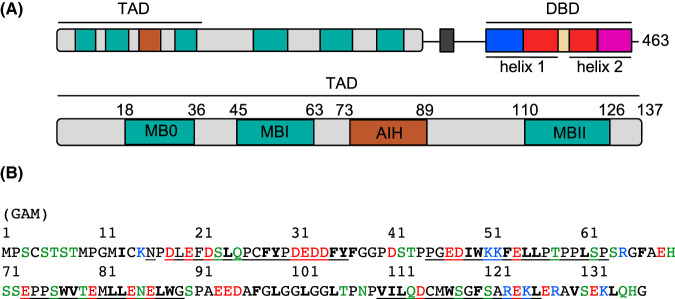
N-myc domain structure and TAD sequence. (**A**) Domain structure of N-myc showing the positions of the structured C-terminal DNA-binding domain (DBD), myc boxes and the N-terminal transactivation domain (TAD). The TAD is magnified below, showing the positions of myc boxes MB0, MBI and MBII and the Aurora-A interaction helix (AIH). (**B**) Primary sequence of the N-myc TAD; hydrophobic residues are shown in bold, basic residues in blue, acidic residues in red, polar residues in green. The positions of the myc boxes and AIH are underlined.

The primary amino acid sequence of N-myc_1-137_ (or N-myc TAD) reveals an amino acid composition typical for an IDP (Figure 1) [68]. For example, the sequence has a comparatively large proportion of Pro (12%), Gly (10%) and Ser (10%) residues, which all disfavour secondary structure formation [69]. The sequence is also enriched in Glu (10%) and Asp (7%) residues, as is typical for TADs [70], but has a low number of Ala (4%), Ile (2%) and Val residues (2%).

**Figure 2.**
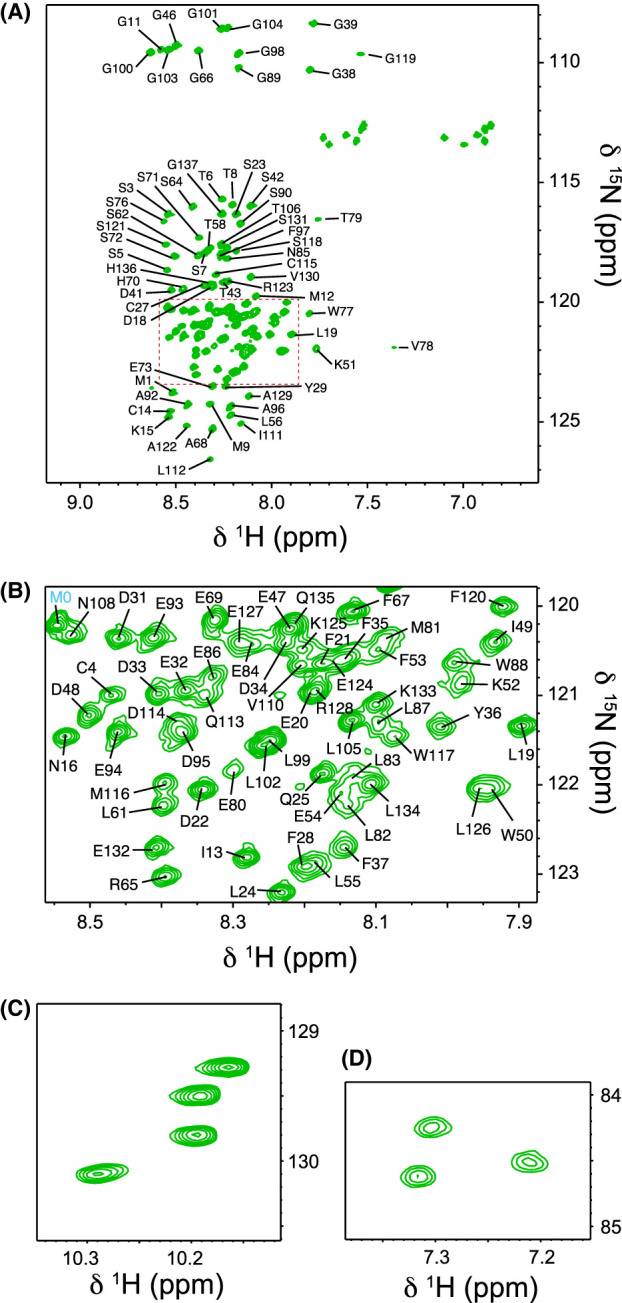
Assigned ^1^H–^15^N HSQC spectrum of the N-myc TAD. (**A**) Overall spectrum (omitting Trp and Arg Hε–Nε correlations). Plot shows the assignment of peaks outside the central region, note some of the weak outlying peaks e.g. Val78, Leu112, Lys51. Gln (ε) and Asn (δ) sidechain NH_2_ peaks (^15^N ∼113 ppm) are unassigned. (**B**) A magnified view of the central region showing the lack of dispersion here as well as variation in the intensity of peaks. (**C, D**) Sidechain Hε–Nε correlations. Four Trp (**C**) and three Arg (**D**) sidechain correlations are resolved accounting for Trp51, Trp77, Trp88, Trp117, Arg65, Arg123, and Arg128.

The ^1^H–^15^N HSQC spectrum of N-myc TAD (Figure 2) shows the low dispersion characteristic of an IDP [71], but uncharacteristically — and as previously observed for c-myc — peaks are not uniformly sharp, exhibiting a range of line-widths, with a subset of broad peaks requiring an increased number of scans to be clearly observed. Usually, IDPs have fast rotational correlation times, resulting in slow *R*_2_, which is realised in the form of sharp peaks. The broad peaks indicate that there are regions that exhibit structure or otherwise altered dynamics within the N-myc TAD sequence (Supplementary Figure S1, particularly Lys51–Leu56, Ser76–Gly89 and Ile111–Ala122). This feature was initially probed by collecting spectra over a range of temperatures (283.1–310.2 K). Most peaks diminish in intensity as the temperature was raised; presumably increased solvent exchange outweighs the benefits of faster tumbling at higher temperatures. Some small regions (close to residue 30, 80 and 110) show increased intensities at moderate temperatures and are slower to lose relative intensity at high temperatures (Supplementary Figure S1B). Plotting CSPs as a function of temperature (Supplementary Figure S1C) identified two regions, one close to Thr58 and the other near Ile111, that stand out as having large temperature coefficients. Both regions have a number of neighbouring prolines. Residues in the region 120–130 have particularly low temperature coefficients, consistent with a level of protection afforded to the H–N bond through hydrogen bonding. A number of peaks for residues in this region moved in the opposite direction (downfield) on increasing temperature compared with peaks for the rest of the protein sequence (which uniformly moved upfield in ^1^H and ^15^N).

Usefully, as a marker of protein integrity, the appropriate number of resolved sidechain resonances were observed for the four Trp Nε–Hε, six Gln (Nε–Hε) or Asn (Nδ–Hδ) pairs and even the three Arg Nε–Hε peaks (Figure 2). N-myc TAD is labile to proteolysis, despite efforts made to purify the sample and use of protease inhibitors, as shown by the emergence of degradation peaks in the ^1^H–^15^N HSQC spectrum within ∼3 days with the sample held at 10°C.

The utilisation of standard BEST versions of ^1^H-detected 3D experiments (HNCO, HNcaCO, HNcoCA, HNCA, HNcocaCB, HNcaCB, HBHAcoNH, Supplementary Table S1) enabled the unambiguous assignment of ∼60% of the N-myc TAD backbone. A few regions however were particularly challenging to assign; for example, residues Ser76–Trp88 were characterised by peak broadening and most peaks were positioned within regions of high ^1^H degeneracy within the ^1^H–^15^N HSQC spectrum causing peak overlap. Full assignment was achieved by recording assignment spectra for truncated sections of N-myc TAD and transferring assignments to the full-length construct to remove ambiguities. A C-terminal truncation of the TAD, N-myc_64–137_, and a fusion of GB1 [55] to N-myc_18–59_, provided excellent overlap with full length ^1^H–^15^N HSQC spectra for peaks away from the termini (Supplementary Figure S2). The shorter constructs yield spectra with fewer N-myc peaks and exhibit more favourable dynamics [72], and thus spectra have enhanced sensitivity and resolution in comparison with N-myc TAD spectra. All told, and coupled with some corroborating ^13^C- and ^15^N-detected spectra (Supplementary Table S1) [73], assignments for all backbone N, CO and Cα resonances within N-myc TAD (and the majority of those for Cβ, Hα and Hβ) were achieved; see BMRB entry 52066.

### N-myc TAD structure and dynamics

**Figure 3.**
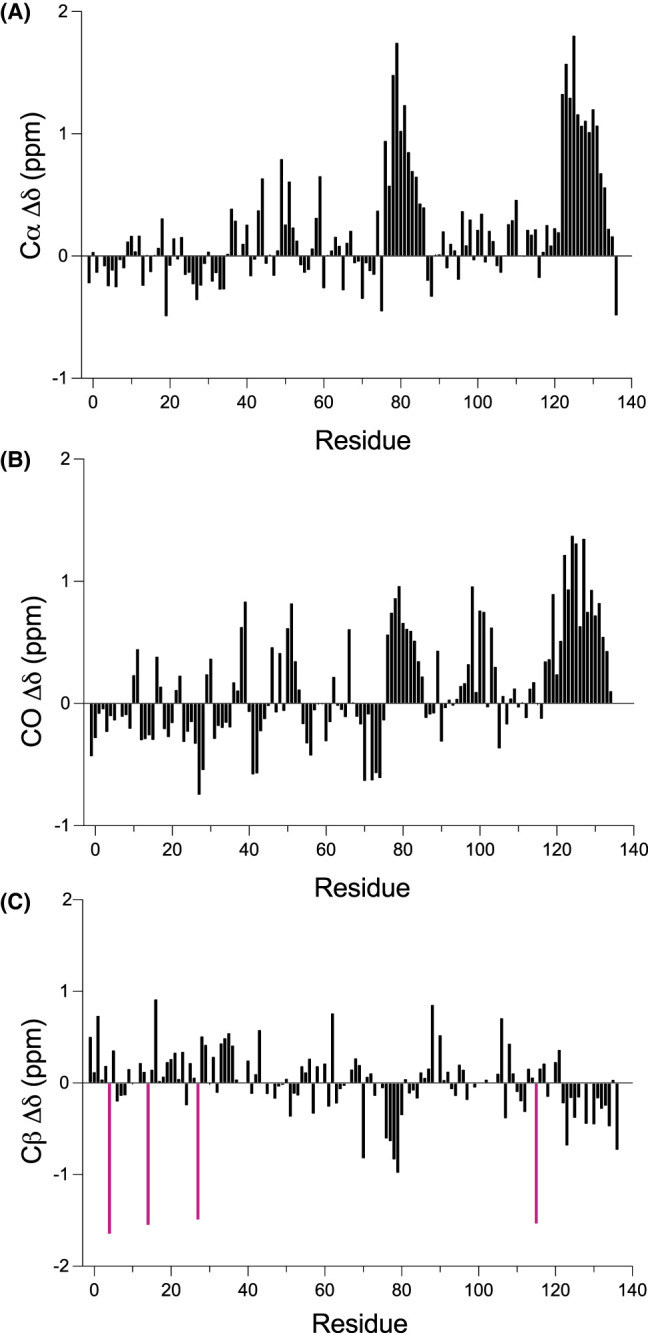
Structural propensities within N-myc TAD. Secondary shifts (Δδ) for Cα (**A**), backbone carbonyl (**B**) and Cβ (**C**) nuclei across the sequence of N-myc TAD. Positive runs of Cα/CO Δδ values indicate α-helices, negative runs of Cα/CO Δδ values indicate β-strands and values nearing zero or with variation in sign of consecutive values indicate a lack of structure. The trends are mirrored for Cβ Δδ values. A comparison of Cα Δδ for N-myc TAD with the truncated variants is shown in Supplementary Figure S3A. The four Cβ Δδ values in purple are for the four Cys residues in the sequence; we contend that the anomalous values are due to an error in the reference coil value for Cys [60].

Secondary chemical shift (Δδ) data — the difference between a measured chemical shift and a reference chemical shift for the same residue in a coil configuration — can be used to indicate structural propensities within proteins [74]. For example, ^13^Cα Δδ values are positive in α-helices and negative in β-strands, whereas ^13^Cβ Δδ values display the opposite trend. In N-myc TAD (Figure 3A), the close-to-zero ^13^Cα Δδ values and weak correlations between adjacent residues indicate that the N-terminal portion of N-myc TAD (aa 1–76) is largely random coil albeit with potentially some weak extended/β-strand propensity in MB0 (Leu24–Asp34) and weak helical propensity in MBI (Asp48–Phe53). There are two regions in the C-terminal portion of N-myc TAD (Trp77–Glu86 and Ala122–Glu132) with consistent positive values of ^13^Cα Δδ indicating that these parts of the protein have helical propensity. This pattern of ^13^Cα secondary shifts is very similar for the truncated GB1-N-myc_18–59_ and N-myc_64–137_ constructs, with the weak strand propensity in MB0 reproduced and consecutive positive secondary shift values shown between Val78–Asn85 and Ala122–Glu132 (Supplementary Figure S3A). Secondary shift patterns for the backbone carbonyl (CO) follow the same trend as those for Cα (Figure 3B), whilst those for ^13^Cβ show inverted features, with regions with helical propensity appearing as negative Δδ values (Figure 3C). The program TALOS-N [61] was used to provide a more general prediction of secondary structure utilizing more fully the backbone chemical shifts along the sequence (Supplementary Figure S3B). As expected, most of N-myc TAD is random coil but again TALOS-N places two α-helices at Trp77–Glu84 and Ala122–Glu132.

**Figure 4.**
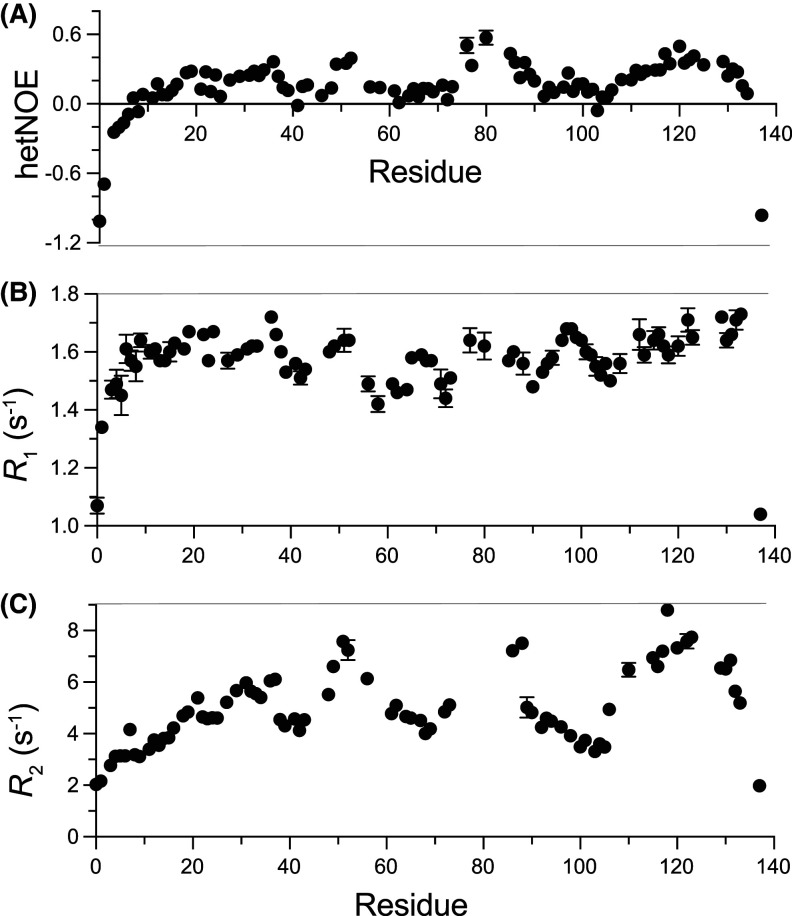
Relaxation measurements for the N-myc TAD. ^1^H–^15^N heteronuclear NOEs (**A**), longitudinal (*R*_1_, **B**) and transverse relaxation rates (*R*_2_, **C**). Experiments were carried out at 10°C. Residues missing from the plots include prolines and overlapping/broad peaks for which intensity changes could not be accurately gauged.

To investigate further the behaviour of the N-myc TAD in the context of local and global structure, a set of three relaxation measurements, ^1^H–^15^N NOE, *R*_1_ and *R*_2_, was employed to probe the local dynamics of the protein. ^1^H–^15^N NOE values inform whether individual residue dynamics on the picosecond to nanosecond (ps–ns) time scale are determined by the overall rotation of the molecule (τ_C_) and are thus ‘fixed’ or rigid in nature, or whether their dynamics is faster than τ_C_, thus indicating a degree of flexibility [75]. Negative NOE values indicate large amplitude motions on short timescales as observed at the N- and C- termini of N-myc TAD (Figure 4A) which have the highest degree of freedom in their motions [76]. Regions with restricted local movements result in increasingly positive NOE values as their rotational motions approach τ_C_. Most residues in N-myc TAD are characterised by ^1^H–^15^N NOE values in the range 0.2–0.3, similar to those observed in c-myc [44], indicating that most of the TAD is disordered. Within the TAD sequence there are stretches of residues which are characterised by higher NOE values (>0.35). These regions (Trp50–Phe53, Trp88–Ser90 and Ser118–Ser121) are likely to experience more restrictive motions of the N–H vectors (Figure 4A) [77].

*R*_1_ and *R*_2_ measurements (longitudinal and transverse relaxation rates, respectively) also report on ps–ns motions while *R*_2_ is also impacted upon by µs–ms time-scale changes [75]. Away from the termini, *R*_1_ values remain relatively consistent (1.4–1.7 s^−1^, average 1.6 s^−1^) across the length of N-myc TAD, with some subtle variation mirroring the hetNOE profile (Figure 4B). This indicates that on the ps–ns time scale residues are broadly similar in their dynamics. *R*_2_ values increase with slower tumbling and are impacted by µs–ms dynamic processes, which signifies more ‘global’ conformational motions such as protein folding, oligomerisation, or chemical exchange between different conformers [78]. The N-myc TAD *R*_2_ values vary across the sequence, ranging between 2.0 and 8.0 s^−1^ with an average of 4.9 s^−1^. Residues with increased *R*_2_ values are Trp50–Phe53, Leu87/Gly89 and Ile111–Glu132, the latter two regions partially align with those shown to exhibit helicity (Figure 3).

### N-myc peptides for regions of interest

**Figure 5.**
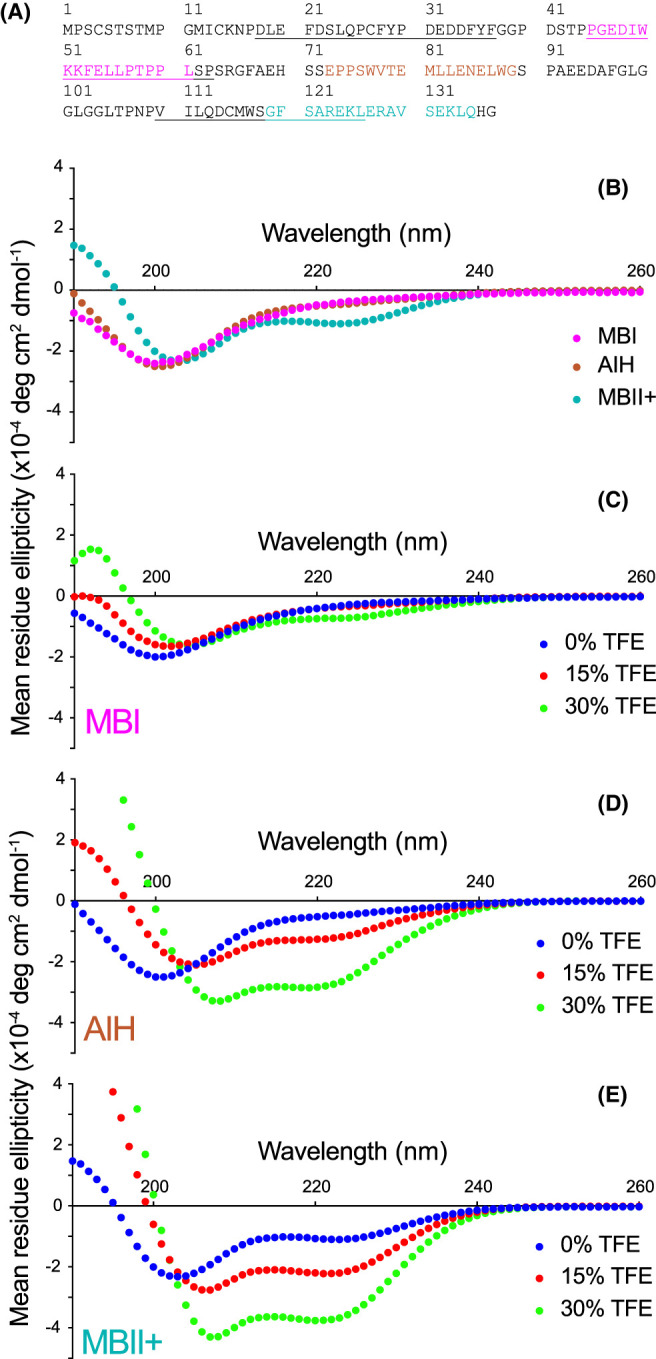
Circular dichroism (CD) spectroscopy of N-myc TAD peptides. (**A**) N-myc TAD sequence with the positions of the peptides coloured and myc box positions underlined. (**B**) Overlaid CD spectra for the three peptides in buffer. (**C–E**) Comparison of CD spectra for the MBI peptide (**C**), AIH peptide (**D**) and MBII+ peptide (**E**) in buffer, 15% TFE and 30% TFE. Experiments were carried out at 5°C.

The NMR data highlight sections of N-myc TAD that behave differently to the rest of the protein, be that through transient structure formation and/or altered dynamics. We selected three equal-length 17-residue sections of N-myc for further structural analysis using CD spectroscopy (Figure 5A). Peptides were synthesized for Pro45–Leu61 (MBI); Glu73–Gly89, the region that appears as a helix in the N-myc–Aurora-A crystal structure [31] or ‘Aurora-A-interacting helix’ (AIH) [79]; and Gly119–Gln135, a region with helical propensity that extends beyond MBII (MBII+). At 5°C, the CD spectra for MBI and AIH appear disordered with a characteristic single minimum in the mean molar residue ellipticity (MRE) at 200 nm [80]. However, MBII+ exhibits a positive band at 190 nm and an increased magnitude negative band at 222 nm (Figure 5B), characteristics of helicity [81]. An estimate of helical content using the MRE_222_ value shows MBII+ to be 20% helical — which is significant helical content for a short peptide [67] — whereas MBI and AIH might be ∼10% helical. The addition of 2,2,2-fluoroethanol (TFE), a known helix promoter [82], showed that whereas MBI has limited potential to form a helix, AIH can be driven to become very highly helical (estimate >70%), nearly on a par with MBII+ under the same conditions. TFE results can be used to mimic how peptides could interact with other proteins. These results suggest that the AIH region, although not natively helical, can be driven to bind in a helical conformation, as observed in its interaction with Aurora-A, perhaps in a bind-and-fold mechanism. MBII+ exhibits a clear helical signature even without the addition of TFE, marking it as a potential site to bind to partner proteins in this structured form.

MBII is important for interaction of myc proteins with many histone acetyl transferase enzymes, at least in part due to its known role in binding the adaptor protein, transformation/transcription domain-associated protein (TRRAP) [25,83]. MBII sequences for N-myc (110–126) and c-myc (128–144) have 76% amino acid identity, but beyond that N-myc includes three additional helix promoting residues ‘ERA’ prior to a conserved ‘VSELK’ motif (Supplementary Figure S4); the ‘VS’ residues have lower helical propensity and likely act to limit helix propagation [84]. CD spectra for a c-myc MBII+ peptide (residues 137–153, Supplementary Figure S4) show it has much weaker helicity (<10% helix) than the equivalent stretch in N-myc. However, as with the AIH in N-myc, the helicity of c-myc MBII+ is significantly enhanced on addition of TFE.

### Interaction with Aurora-A

**Figure 6.**
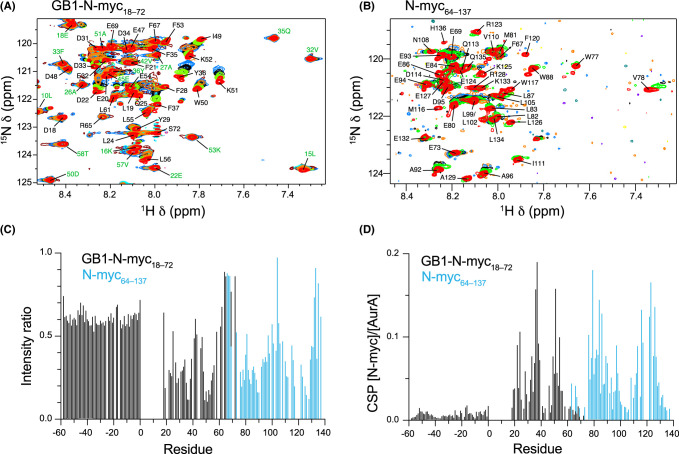
NMR titration experiments for Aurora A kinase domain with N-myc TAD truncates. (**A**) ^1^H–^15^N HSQC spectrum of GB1-N-myc_18–72_ (red) and then after addition of increasing amounts of Aurora A kinase domain (lime green, black, sky blue, orange). (**B**) ^1^H–^15^N HSQC spectrum of N-myc_64–137_ (red) and then after addition of increasing amounts of Aurora A kinase domain (lime green, black, sky blue, orange). (**C**) Ratio of intensities at a [Aurora A]:[N-myc] molar ratio of 0.2:1 compared with unbound N-myc truncates. The GB1 sequence is renumbered as negative numbers. (**D**) Changes in chemical shift perturbations as a linear function of [Aurora A]:[N-myc].

N-myc TAD is a known binder of the Ser/Thr-kinase Aurora-A [85]. Here, NMR was used to assess the interaction of the full N-myc TAD with Aurora-A. Addition of small volumes of concentrated Aurora-A to a ^15^N-labelled sample of N-myc TAD at 25°C led to loss in ^1^H–^15^N HSQC spectral quality (Supplementary Figure S5). This indicates that, as expected, the two species interact extensively — peak intensity losses in N-myc come about for sites of interaction with Aurora-A due to loss in dynamic freedom (and changes in *R*_2_). From the spectrum of N-myc TAD in complex with Aurora-A, isolated peaks for some residues are completely lost (e.g. Val78, Thr79), strongly indicating them as sites of interaction. However, the initial peak overlap and poor quality of the N-myc–Aurora-A complex spectrum prevented a more thorough analysis. More useful information was gained by running the same Aurora-A NMR titration with ^15^N-labelled truncates of N-myc (Figure 6). As with the assignment strategy, by dividing the TAD into two sections, the peak overlap is reduced allowing more residues to be individually assessed. It also has the benefit of reducing the overall extent of interaction between Aurora-A and each N-myc truncate and thus the change in *R*_2_ dynamics on binding.

A slightly longer ^15^N-labelled N-myc fusion protein (GB1-N-myc_18-72_) was used rather than the assigned GB1-N-myc_18-59_, as this provided some overlap with the C-terminal N-myc_64–137_ construct. Assignment of the C-terminal part of GB1-N-myc_18–72_ was straightforwardly achieved through comparison with full length. On addition of Aurora-A not only peak intensity changes but CSPs were observed (Figure 6A,B). Plotting the ratio of peak intensities for spectra with Aurora-A present to those in initial spectra shows there is a general loss in peak intensity across N-myc samples sequences, but strongly binding regions suffer more significant losses (Figure 6C). The GB1 peaks provide a useful control since, despite not being involved in the interaction, the overall change in protein tumbling on binding means that their intensities drop to ratios that are consistently ∼0.6 across its sequence. Interestingly, there are regions within the N-myc sequence that maintain significantly higher intensity ratios than in GB1, notably Ser64–Ala68, Gly104 and Glu132–Gln135 at the C-terminus. It is possible that, as seen with c-myc binding to Bin1 [44], these regions are slightly liberated from dynamic intramolecular interactions when neighbouring parts of the sequence are occupied in binding to Aurora-A. There appear to be four regions within N-myc with the most significant intensity drops: Leu19–Gly39 (MB0), Asp48–Leu56 (MBI), Ser76–Ser90 (AIH), Val110–Ala129 (MBII). The first three of these regions are known interactors, with Ser76–Gly89 visible in the Aurora-A–N-myc crystal structure [31]. The last region, which aligns with MBII has not been previously characterised, and so the data indicate that the N-myc–Aurora-A interface is more extensive than previously thought. The patterns of CSPs observed on binding mirror those of the intensity drops with non-interacting sites (and GB1) showing small shifts and larger CSP observed for the regions described above (Figure 6D). The CSP plot shows two peaks within the Leu19–Gly39 region, which could suggest that there are two distinct binding motifs herein separated by a flexible linker. Expanding on our previous work [31], we performed coprecipitation experiments to show the interaction of Aurora-A kinase domain with biotinylated N-myc peptides (Supplementary Figure S6). This confirmed the binding of a MBII-containing peptide (residues 107–137) to Aurora-A, albeit the weak band indicates it has significantly lower affinity than the previously known sites (N-myc 18–47 and N-myc 61–89).

### *In vitro* phosphorylation of N-myc TAD

**Figure 7.**
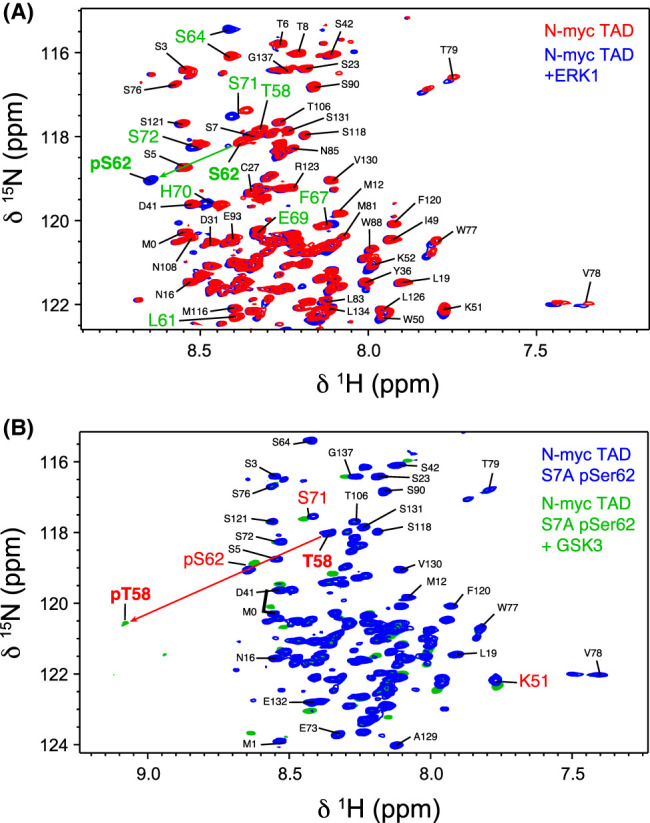
*In vitro* phosphorylation of N-myc TAD followed by NMR. (**A**) ^1^H–^15^N HSQC spectrum of N-myc TAD (red) and then after addition of ERK1 (blue). (**B**) ^1^H–^15^N HSQC spectrum of N-myc TAD S7A, pre-phosphorylated by ERK1 (blue) and then after addition of GSK3 (green).

Phosphorylation of the TAD is associated with regulated ubiquitination–proteolysis of N-myc, and NMR provides a means to monitor the individual phosphorylation events catalyzed by different kinases. ERK1 is a proline-directed kinase which becomes stimulated by mitogenic signals and has been shown to specifically phosphorylate Ser62 in c-myc, both *in vivo* and *in vitro* [34,86]. The ^1^H–^15^N HSQC spectra of N-myc TAD prior to addition and post-incubation with 0.3 µM of ERK1 were compared (Figure 7A). The ^1^H–^15^N peak for Ser62 undergoes a large CSP characteristic of phosphorylation (downfield shift in both ^1^H and ^15^N) [64]. Peaks for residues surrounding this site: Leu61, Ser64, Arg65 and Gly66 also experience significant CSPs, indicating that their chemical environment has changed, due to the presence of the nearby phosphoryl group. Small CSPs were also observed for more distant His residues and their nearest neighbours (His70, Leu71, His136, Gly137). These changes are most likely not associated with ERK1 activity directly but are due to changes in protonation state of the histidines as the pH in the solution subtly decreases due to ATP hydrolysis.

Four additional Ser/Thr residues within N-myc TAD (Thr43, Thr58, Ser90, Thr106) meet the criteria for the ERK1 minimal consensus sequence ([Ser/Thr]–[Pro]) [87]. We therefore examined if these sites were also targeted. Thr43 and Thr106 also undergo phosphorylation by ERK1, albeit on a much slower timescale than Ser62 (Supplementary Figure S7A). Unlike Ser62, which becomes fully phosphorylated within the time required to collect the spectrum, Thr43 and Thr106 take on the order of 10 and 5 h, respectively, for their HSQC peaks to diminish to 50% of the original intensity. There was concomitant slow emergence of new, weak, downfield shifted ^1^H–^15^N HSQC peaks associated with these phosphorylated-species. The rate of Thr43 and Thr106 phosphorylation is increased when the experiment is performed at higher temperature (Supplementary Figure S7B), but Ser62 is still by far the favoured site for ERK1 phosphorylation. Interestingly, the Ser90 peak did not appreciably change over the course of phosphorylation experiments, behaving in a manner equivalent to other Ser residues that do not meet the minimal consensus sequence (e.g. Ser23, Ser118, Ser131). The direct effect of ERK1 on Thr58 was difficult to observe since a small CSP from the phosphorylation of Ser62 causes the ^1^H–^15^N HSQC peak of Thr58 to overlap with that of Ser7. This unfortunate feature also prevented the next step in the myc degradation pathway — phosphorylation of Thr58 by GSK3 — from being unambiguously tracked using the wild-type N-myc TAD protein.

To address the peak overlap problem, an N-myc TAD construct incorporating a S7A mutation was produced. The ^1^H–^15^N HSQC spectrum of N-myc TAD^S7A^ overlaps very well with WT N-myc TAD, except for the mutation site itself and peaks for a few surrounding residues (Supplementary Figure S8). Importantly, the absence of the Ser7 peak meant that Thr58 could be independently tracked throughout. N-myc TAD^S7A^ was subjected to the same initial phosphorylation procedures, phosphorylation with ERK1, followed by addition of 0.3 µM GSK3 monitored using NMR (Figure 7B). The shifted Thr58 peak after Ser62 phosphorylation did not show obvious loss in peak intensity (Supplementary Figure S7C) suggesting that Thr58 in N-myc is not efficiently phosphorylated by ERK1, at least in the presence of a neighbouring Ser62/p-Ser62. After addition of GSK3 the Thr58 peak is rapidly and completely lost (Figure 7B, Supplementary Figure S7C), and a new broad peak appears downfield in ^1^H and ^15^N, within the region expected for phospho-species [64]. Some neighbouring residues (e.g. Leu56, Leu61) displayed small CSPs, generally smaller than those observed with the priming phosphorylation of Ser62 (Figure 7).

The action of GSK3 appears to be specific to Thr58 after the phosphorylation of Ser62 with ERK1. We tested the effect of treating unphosphorylated N-myc TAD with GSK3, through following spectral changes with NMR. We confirmed that phosphorylation of Thr58 by GSK3 requires the prior phosphorylation of Ser62, since these peaks were unaffected. To our surprise, however, we observed slow phosphorylation specifically of Thr106 (Supplementary Figure S9). This effect was masked in the presence of ERK1, which also slowly targets Thr106. It appears that this position can be phosphorylated *in vitro* by GSK3 without the need for (*i* + 4) priming phosphorylation, an unusual but not unknown feature for this kinase [88,89]. Intact mass spectrometry (Supplementary Figure S10) also showed low levels of phosphorylated material (+80 Da) were present after direct treatment of N-myc TAD with GSK3.

### Doubly phosphorylated N-myc TAD binds the Fbxw7–Skp1 complex

**Figure 8.**
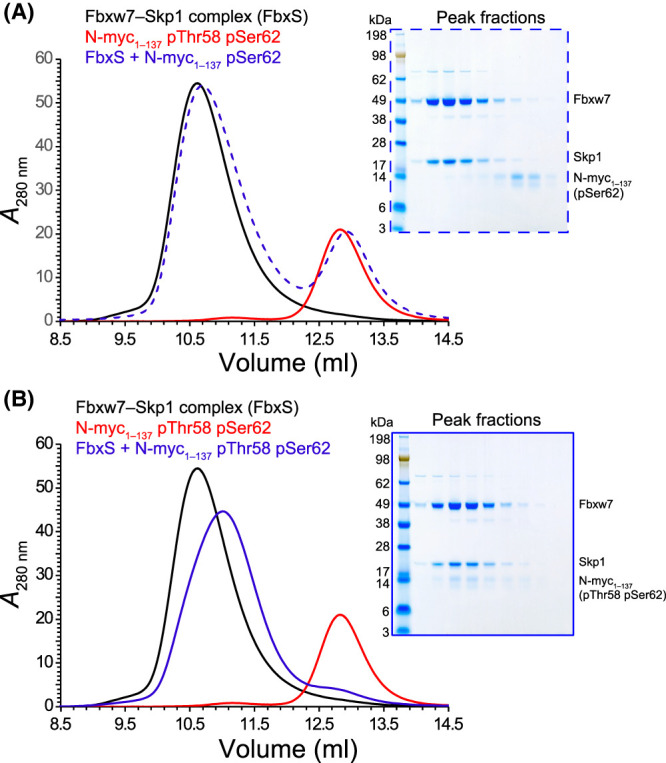
Doubly phosphorylated N-myc TAD (pSer62, pThr58) binds to the Fbxw7–Skp1 complex. Analytical SEC using a Superose12 10/300 GL column was used to assess complex formation between the Fbxw7–Skp1 complex and either singly phosphorylated (pSer62) N-myc TAD (**A**), or doubly phosphorylated (pSer62, pThr58) N-myc TAD (**B**). The chromatogram for the complex is shown in blue, while the chromatograms for N-myc TAD alone or Fbxw7–Skp1 alone are shown in red and black, respectively. 0.5 ml fractions were taken at the same positions in both runs and analysed by SDS–PAGE.

The phospho-degrons of c-myc and N-myc are recognized by Fbxw7–Skp1, which mediates their ubiquitination, targeting them for proteasomal degradation [35,41]. To establish the importance of individual PTMs on Fbxw7–Skp1 recognition, N-myc TAD pSer62 and N-myc TAD pSer62 pThr58 were generated by *in vitro* phosphorylation using ERK1 and GSK3. The reactions were monitored using NMR as described above and stopped by snap freezing in liquid nitrogen. N-myc TAD pSer62 and N-myc TAD pThr58 pSer62 were incubated with Fbxw7–Skp1 and analytical SEC was employed to ascertain complex formation (Figure 8). N-myc TAD pSer62 did not form a stable complex with Fbxw7–Skp1 and the proteins eluted separately at the same elution volumes as individual proteins (Figure 8A), as confirmed by SDS–PAGE. The interaction between the two species only became apparent when N-myc TAD was di-phosphorylated on Thr58 and Ser62. The N-myc peak almost completely disappears and is found to co-elute with Fbxw7–Skp1 (Figure 8B). The interaction between Fbxw7–Skp1 and a pThr58 pSer62 N-myc peptide (residues 47–66) was recently shown to have an affinity <10 nM [37]. Interestingly, the complex between Fbxw7–Skp1 and N-myc TAD pThr58 pSer62 elutes later than the Fbxw7–Skp1 complex alone, suggesting that the interaction with N-myc TAD makes the complex more compact despite an increase in its molecular mass.

**Figure 9.**
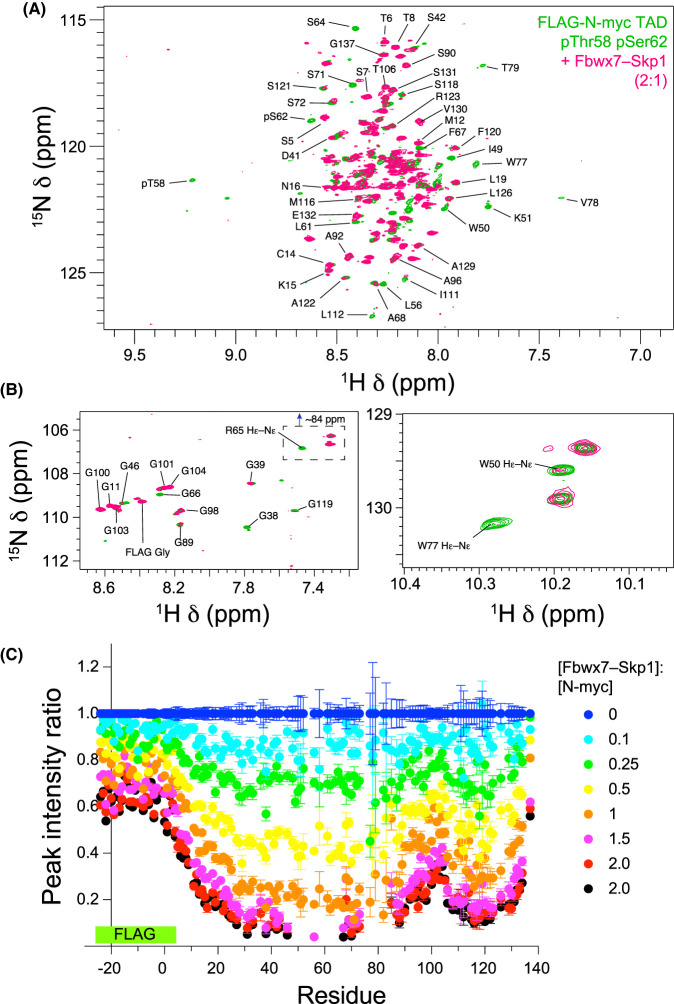
NMR titration of the Fbxw7–Skp1 complex with *in situ* generated doubly phosphorylated N-myc TAD (pSer62, pThr58). (**A**) The central backbone region of the ^1^H–^15^N HSQC spectrum before (green) and after addition of Fbxw7–Skp1 (pink). (**B**) Magnified views of the Gly region (left) and Trp Hε–Nε region (right) of the ^1^H–^15^N HSQC spectrum. Inset is an aliased region (from 84 to 85 ppm) showing Arg Hε–Nε correlations. Peaks that disappear highlight the parts of the sequence that interact most strongly. (**C**) Ratio of peak intensities for each residue as a function of increasing molar ratio of [Fbxw7–Skp1]:[N-myc].

NMR was again used to map the interaction of Fbxw7–Skp1 on doubly-phosphorylated N-myc. N-myc TAD pThr58 pSer62 was generated through in-tube treatment with a mixture of ERK1 and GSK3. Further kinase activity was then quenched by addition of EDTA. Small volumes of concentrated Fbxw7–Skp1 were added to the sample which brought about loss in peak intensities in ^1^H–^15^N HSQC spectra (Figure 9A,B). No CSPs were observed, and the effect on intensity is unsurprisingly broad given the size of the Fbxw7–Skp1 complex (70 kDa). The major peak intensity loss is centred on the phosphodegron site at pT58 (Figure 9C). An unexpected second site of potential interaction was revealed around MBII. This region shows more pronounced peak intensity losses than the neighbouring sites of the Gly-rich region (∼Gly100) and the C-terminus. Fbxw7–Skp1 titrations with singly-phosphorylated (pSer62) and unphosphorylated N-myc TAD also showed clear peak intensity reductions (Supplementary Figure S11). Thus, Thr58 phosphorylation is not required for the proteins to interact, but it does significantly raise the affinity to a level required for a complex stable to be observed in SEC. From NMR, the MBI phosphodegron site is the core of the interaction for N-myc TAD pThr58 pSer62. All peaks local to Thr58 (including local sidechain correlations for Trp50 and Arg65) are completely lost once the molar ratio exceeds 1:1 (Figure 9). In contrast, singly-phosphorylated (pSer62) and unphosphorylated N-myc TAD retain peak intensity close to MBI (Supplementary Figure S11). Changes in peak intensity away from MBI vary more sharply, likely as a result of fast exchanging, weak interactions of Fbxw7–Skp1 with MBs and the AIH. Peak intensity changes close to MBII appear to be largely independent of phosphorylation state. As with the interaction with Aurora-A, secondary binding sites on N-myc are involved, adding further complexity to the interaction of N-myc TAD with Fbxw7–Skp1.

## Discussion

Myc TADs are intrinsically disordered regions, vital for the regulation of gene expression, and essential for the oncogenic functions observed in myc proteins. Both N-myc and c-myc interact with a large number of proteins via the TAD to mediate these functions. This region of N-myc also contains the MBI phosphodegron, which is an important determinant of protein stability, and a site which is frequently mutated in N-myc driven cancers [90]. Structural understanding of these regions has been limited to crystal structures of small fragments and NMR studies on an N-terminal portion of c-myc. The assignment of the full TAD of N-myc will be useful to the field for understanding the dynamics and interactions which underpin the oncogenic and physiological functions of this part of N-myc. In this study, we have characterised the dynamics of N-myc in solution, expanded the known interaction region with two key binding partners and explored the phosphorylation of the MBI phosphodegron.

### Multi-site interaction with Aurora-A perturbs the N-myc TAD

We have previously mapped the interaction between N-myc and Aurora-A using truncated N-myc fragments and determined a crystal structure of N-myc_28–89_ bound to the kinase domain of Aurora-A [31]. Only residues 61–89 were observed in the crystal structure, with residues 76–89 forming an α-helix packed onto the C-lobe of Aurora-A. This region of N-myc matches one of the areas with intrinsic helical propensity shown in the NMR analysis above, suggesting there is a level of ‘templating’, i.e. the intrinsic helicity of this region and Aurora-A binding are coupled, although it is difficult to say which feature dominates. The N-terminal section of N-myc was not observed in the crystal structure although FP binding assays showed that N-myc_18–47_ could bind independently to Aurora-A.

NMR studies on this interaction showed the expected changes in ^1^H–^15^N resonances in MB0 and the 74–89 helical region, both in terms of intensity and chemical shift. The perturbation of residues close to, or in, MBII was surprising. This may represent a new N-myc–Aurora-A interaction interface which acts in concert with the other two sites. While it is common for myc proteins to bind to their partners using more than one linear motif [30,53,91], the use of three motifs spanning across ∼120 residues indicates a greater degree of multivalency than was previously thought. The interaction with Fbxw7–Skp1 also involves a broader region of the TAD than the canonical phospho-degron. Taken together, these results highlight the value of using NMR to probe these interactions in the context of extensive regions of N-myc and other IDR proteins, to complement methods based on the use of peptide fragments.

### Two-site, specific phosphorylation of N-myc MBI

Using in-NMR kinase assays, we established the strong preference of ERK1 for Ser62 and the dependence of GSK3 on this priming event for efficient and selective phosphorylation of Thr58. Phosphorylation of Ser62 and subsequently double phosphorylation of Ser62 and Thr58 allowed for unambiguous assignment of the ^1^H–^15^N resonances of the phosphorylated residues. The absence of CSPs other than in MBI is strongly suggestive that there are not any major changes in N-myc TAD structure driven by either phosphorylation event. This was not entirely unexpected, given that phosphorylation at Ser62 in c-myc 1–88 had no major effects on the chemical shifts outside of MBI either. That this doubly phosphorylated material forms a stable complex with Fbxw7–Skp1 corroborates the findings of Welcker and colleagues [37] who found that doubly phosphorylated MBI was the predominant c-myc species found bound to Fbxw7 in cells and additionally determined the structure of a doubly phosphorylated MBI peptide in complex with Fbxw7–Skp1.

The N-myc TAD is thought to be post-translationally modified on at least three other sites. Two of these sites are close to the MBI phosphodegron; S64 which is phosphorylated and R65 which is thought to be methylated [92,93]. A site close to MBII, K133, is also thought to be acetylated [94]. The approach taken to monitor phosphodegron phosphorylation here can also be used to assess post translational modifications on these other sites, either to determine their potential roles in changing phosphorylation dynamics of MBI, in changing N-myc TAD dynamics, or in interacting with binding partners.

### Ordered regions of N-myc TAD beyond the myc boxes participate in protein–protein interactions

Analysis of the solution dynamics of N-myc TAD indicate that is not a fully disordered polypeptide: in several regions of the TAD, its motions are less dynamic on the ps–ns timescale than would be expected for a fully disordered polypeptide, resulting in heterogeneity in peak intensities across the TAD in NMR experiments. Regions of N-myc TAD involved in protein–protein interactions (notably AIH and MBII) have clear helical propensity, and the protein samples these secondary structure conformations in solution. We do not yet understand the consequences of this conformational sampling, however for c-myc it has been observed that binding of Bin1 to one site increased flexibility elsewhere in the protein [44]; similar behaviour was observed here when N-myc binds Aurora-A. Molecular dynamics simulations of both c-myc 1–88 and the full-length c-myc–Max complex also suggest that myc can sample many conformations and that conformational sampling in their TAD may be a viable target for drugging c-myc [95]. It remains to be determined if the constrained dynamics of myc proteins have roles in myc biology. However, this feature of constrained dynamics at the ps–ns timescale is not observed in all intrinsically disordered TADs. A well-studied example in NMR is that of the human p53 TAD. This appears to be highly dynamic, as demonstrated by its hetNOE values which are at, or below, zero [96,97].

Chemical shift data indicate that N-myc TAD has helical propensity in residues 77–86 inclusive and 122–132 inclusive, but the equivalent sequence in c-myc has yet to be characterised by NMR [33] (Supplementary Figure S12A). We therefore used AlphaFold2 and AGADIR to predict and compare the secondary structure characteristics of this region in the two proteins (Supplementary Figure S12B–D). The AGADIR algorithm predicts the helical propensity based on CD data from monomeric peptides [98]. In contrast AlphaFold2 is based on information from experimental structures including protein complexes, and can identify conditional folding, particularly of helices, in IDRs [99]. Both methods predict helices at the C-terminus of the TAD (MBII+), albeit more confidently for N-myc than c-myc (Supplementary Figure S12D,E). CD demonstrated that the N-myc MBII+ peptide has significant helical content in solution even in the absence of TFE, suggesting that it may not need significant folding-upon-binding to interact as a helix. In contrast, c-myc MBII+ has negligible helicity in the absence of TFE. The first five residues of MBII+ are in the highly conserved MBII region of the protein, with ∼3 helical turns beyond the MBII region that could act as a single unit for interaction with binding partners. We could therefore consider the functional unit of MBII to extend into this helix, comprising 110–137 in N-myc but to varying degrees in other myc homologues. The AIH of N-myc, and its equivalent in c-myc are confidently predicted by AlphaFold2, but AGADIR predicts low helical propensity for N-myc and virtually none for c-myc (Supplementary Figure S12D,E). While the chemical shift data indicate that the AIH is sampling helical conformations in solution, CD data for the AIH peptide indicate that there are very low levels of helicity in solution prior to the addition of TFE. Nevertheless, the crystal structure of N-myc bound to Aurora-A kinase demonstrated that residues 75–89 bind to the kinase in an α-helical conformation. Moreover, the interaction with Aurora-A can be reinforced using helix-constrained N-myc AIH peptides [79], suggesting that a level of pre-organization can facilitate binding. A region in c-myc in a similar position to the N-myc AIH, predicted as a helix by AlphaFold2 but not by AGADIR, has been crystallised interacting as a helix with the TBP–TAF1 complex (c-myc 97–107) [30]. Therefore we conclude that significant folding upon binding is required for this region of c-myc or N-myc to interact as a helix. The helices share a ΦVTEΦL sequence pattern (Supplementary Figure S12A), but c-myc lacks the two Trp residues (Trp77 and Trp88) that are important for the N-myc–Aurora-A interaction [31]. These sequence differences allow for diversification of interactions specialised to each myc paralog; different partners can bind to equivalent sites in different myc proteins (e.g. TBP–TAF1 for c-myc and Aurora-A for N-myc).

## Data Availability

Chemical shift data for GB1-N-myc_18–59_, N-myc TAD, and N-myc_64–137_ were deposited in the Biological Magnetic Resonance Data Bank (BMRB) entries 52047, 52066 and 52067, respectively. https://bmrb.io.

## Open Access

Open access for this article was enabled by the participation of University of Leeds in an all-inclusive *Read & Publish* agreement with Portland Press and the Biochemical Society under a transformative agreement with JISC.

## Abbreviations

CD: circular dichroism
CSP: chemical shift perturbation
IDP: intrinsically disordered protein
MRE: molar residue ellipticity
NMR: nuclear magnetic resonance
SEC: size-exclusion chromatography
TAD: transactivation domain
TCEP: tris(2-carboxyethyl)phosphine
