# Supplementary material for: Exploring the dynamics and interactions of the N-myc transactivation domain through solution nuclear magnetic resonance spectroscopy
Source: Biochem J. 2024 Oct 21;481(21):1535–56. doi: 10.1042/BCJ20240248 (PMC11555651; doi:10.1042/BCJ20240248)
Supplement: Supplementary Material [file BCJ-481-1535-s1.pdf]

Exploring the dynamics and interactions of the N-myc transactivation domain through solution NMR

Ewa Rejnowicz,[‡] Matthew Batchelor,[‡] Eoin Leen, Mohd Syed Ahanger, Selena G. Burgess, Mark W. Richards, Arnout P. Kalverda, Richard Bayliss*

[1] Astbury Centre for Structural Molecular Biology, School of Molecular and Cellular Biology, Faculty of Biological Sciences, University of Leeds, Leeds LS2 9JT, UK

*** To whom correspondence should be addressed.**

SUPPLEMENTARY INFORMATION

Table S1. List of NMR experiments for each sample with temperature and spectrometer used.

Spectrum	N-myc$_{1–137}$ (TAD)		GB1-N-myc$_{18–59}$		N-myc$_{64–137}$		GB1-N-myc$_{18–72}$	
	Temp. (°C)	Spec.	Temp. (°C)	Spec.	Temp. (°C)	Spec.	Temp. (°C)	Spec.
^{1}H-^{15}N-HSQC	10–37	750<br>600<br>950	15	600	10	750	15, 37	600
HNCO	10	750	15	600	10	750[a]		
HNcaCO	10	750	15	600	10	750[a]		
HNcoCA	10	750	15	600	10	750[a]		
HNCA	10	750	15	600	10	750[a]		
HNcocaCB	10	750	15	600	10	750[a]		
HNcaCB	10	750	15	600	10	750[a]		
^{1}H-^{13}C-HSQC	10	750	15	600				
HBHAcoNH	10	750	15	600				
CON (C-det.)[b]	10	950						
hCACO (C-det.)[b]	10	950						
CAnCO (C-det.)[b]	10	950						
CAN (N-det.)[b]	10	950						
^{15}N T1	10	600						
^{15}N T2	10	600						
^{15}N–[^{1}H]-hetNOE	10	600						
^{1}H–^{15}N-HSQC Aurora A titration	25	750			35	750	37	750
^{1}H–^{15}N-HSQC ERK1, GSK3 phosphorylation	10–20	600, 950[c]						
^{1}H–^{15}N-HSQC Fbwx7–Skp1 titrations	10	950[c]						

[a] Recorded using 25% non-uniform sampling (NUS). Reconstructed using NMRpipe.
[b] A suite of 2D ^{13}C-detected spectra (CON, haCACO and haCAnCO) and ^{15}N-detected spectra (haCAN) which avoid reliance of the congested ^{1}H dimension were also collected for the N-myc TAD construct. This corroborated the assignment and provided additional ^{15}N, ^{13}CO and ^{13}Cα shifts for proline residues.
[c] 3xFLAG-N-myc$_{1–137}$ (^{15}N-labelled) was used for these experiments.

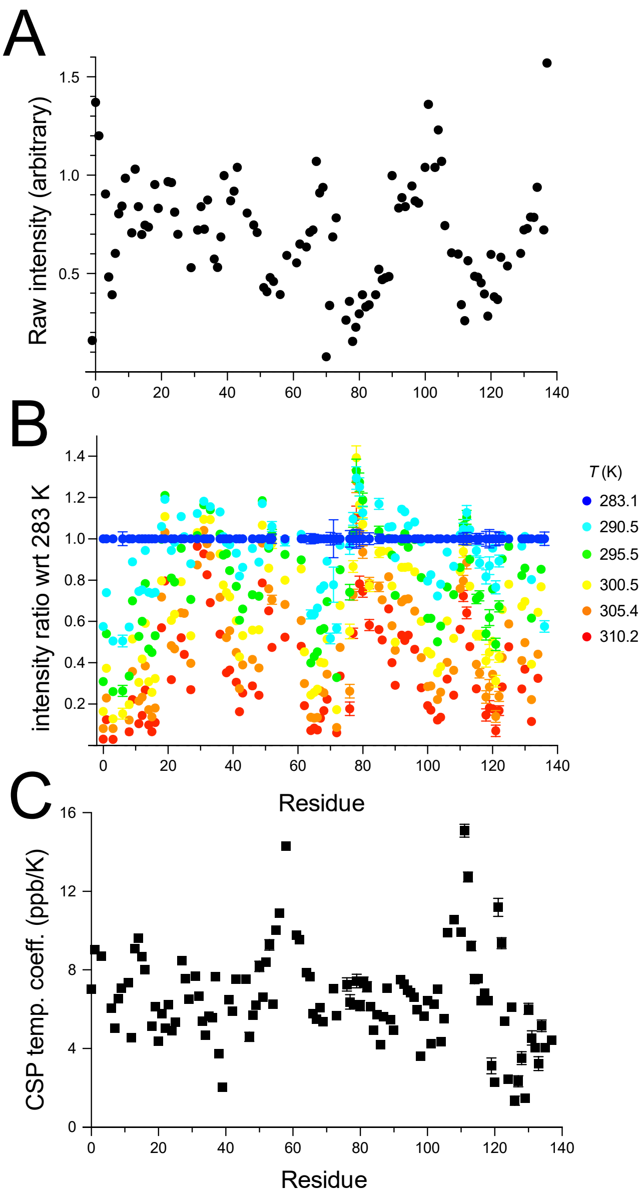

Figure S1. Meta-analysis from $^{1}H–^{15}N$ HSQC spectra of N-myc TAD. (A) Raw intensity measurements for peaks across the sequence. Comparatively low intensity (broad) peaks were observed for Lys51–Leu56, Ser76–Gly89 and Ile111–Ala122 regions. (B) Peak intensities as a function of temperature, plotted as a ratio to the peak intensity at 10 °C. (C) Temperature coefficients for HSQC peaks. The plot shows the change in peak position (or chemical shift perturbation, CSP) as a function of temperature.

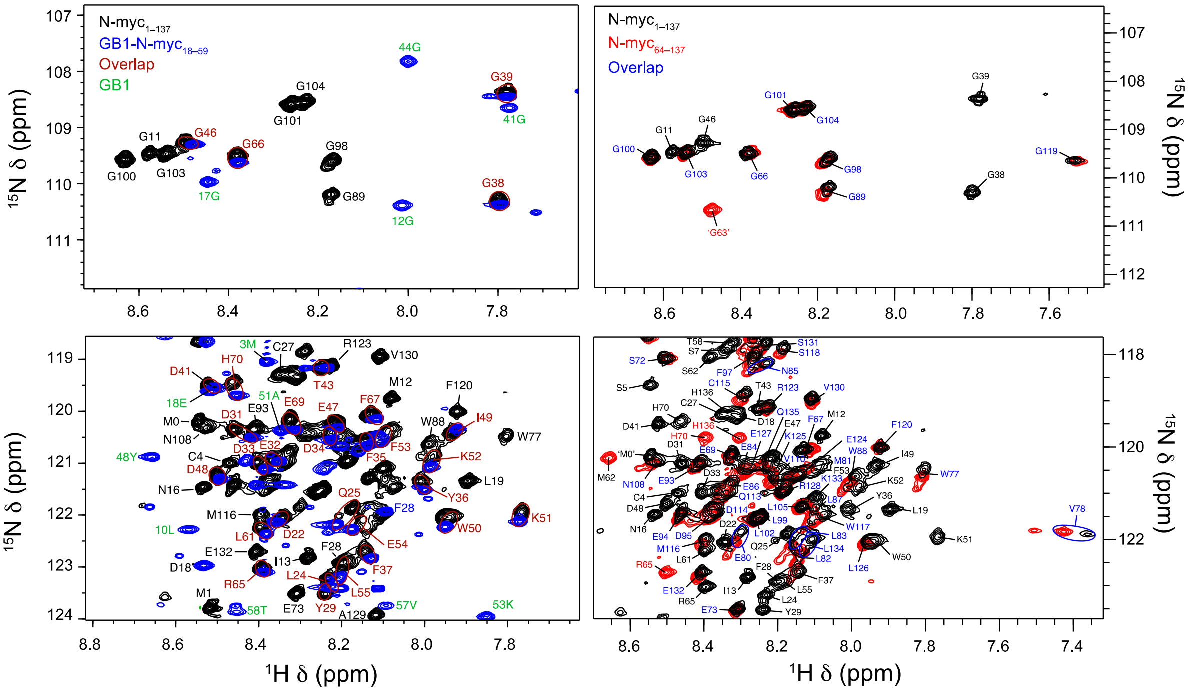

Figure S2. A comparison ^{1}H–^{15}N HSQC spectra of N-myc TAD and the two truncated variants GB1-N-myc$_{18–59}$ (left) and N-myc$_{64–137}$ (right). The upper plots show the glycine region of the spectrum and the lower plots show part of the central region. There are the additional peaks from GB1 in the truncated fusion, but these have greater dispersion and generally do not interfere with the N-myc peaks. Peak position differences are also observed close to the termini in both cases, as would be expected. Small uniform differences in peak resonant frequencies, especially for pH-sensitive His residues, could be due to small variations in buffer. For the GB1-N-myc$_{18–59}$ comparison, peaks from the GB1 part of the sequence are labelled in green; overlapping peaks within the 18–59 sequence are labelled in red. For the N-myc$_{64–137}$ comparison, overlapping peaks within the 64–137 sequence are labelled in blue.

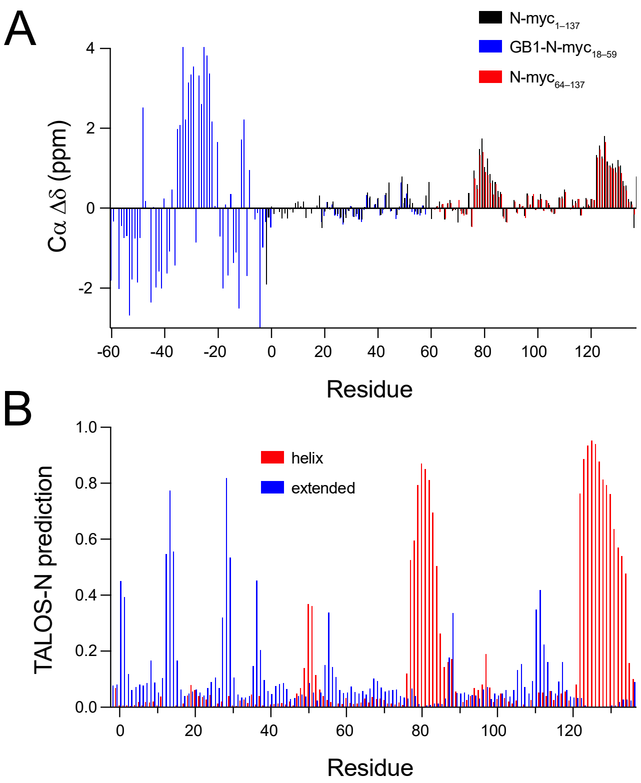

Figure S3. (A) A comparison of the Cα secondary shifts for N-myc TAD (black) and the truncated variants GB1-N-myc$_{18–59}$ (blue) N-myc$_{64–137}$ (red). The overlap is excellent in both cases indicating that the structural propensities are likely to the same in the truncated versions. Note particularly the matching of the weak extended propensities between residues 20–35 for GB1-N-myc$_{18–59}$ and the matching helical propensity regions for N-myc$_{64–137}$. It is also worthwhile comparing the secondary shifts observed in N-myc with the fully-folded helix and β-strands in GB1; for instance, the GB1 helix has Δδ values 3–4 times those in the regions with helical propensity in N-myc. (B) TALOS-N prediction of secondary structure with blue bars corresponding to residues with extended/strand propensities and red bars corresponding to those with helicity.

```
TPNPVILQDCMWSGFSAREKLERAVSEKLQHG   137 N-myc
FIKNIIIQDCMWSGFSAAAKL   VSEKLASYQ  153 c-myc
     I QDCMWSGFSA A   KL   VSEKL       Ident.
```

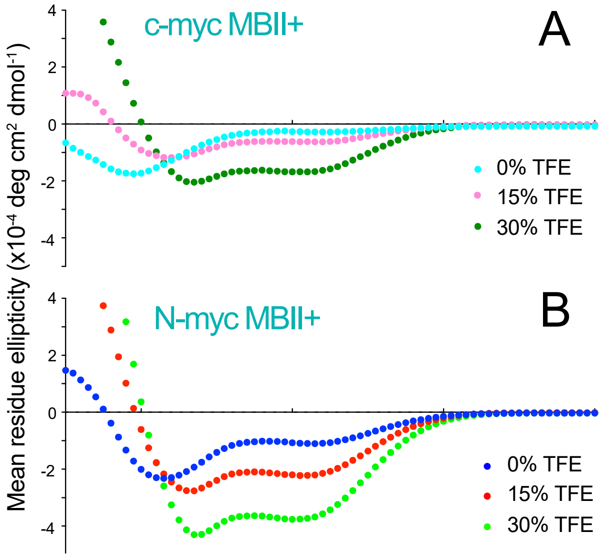

Figure S4. Circular dichroism (CD) spectroscopy of c-myc MBII+ peptide. Sequence alignment for the MBII (underlined) and MBII+ regions (coloured teal) in N-myc and c-myc. (A) Comparison of CD spectra for the c-myc MBII+ peptide in buffer, 15% TFE and 30% TFE. (B) To facilitate comparison, spectra for the N-myc MBII+ (already shown in Fig. 5E) are repeated here. Experiments were carried out at 5 °C.

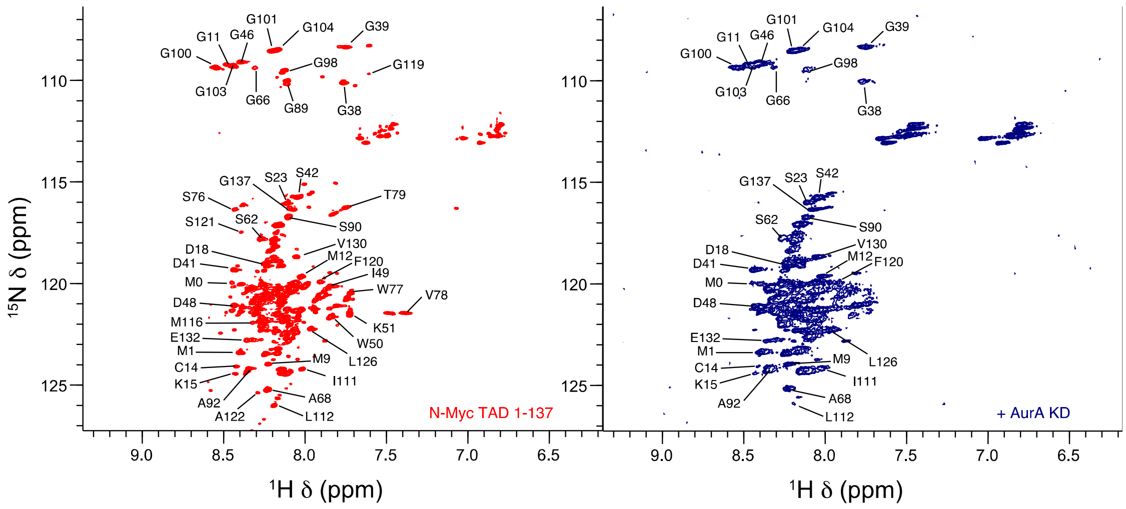

Figure S5. ^{1}H–^{15}N HSQC spectrum for N-myc TAD before (red) and after addition of Aurora A kinase domain (navy blue). Specific peak loss is observed but there was significant attenuation of spectral quality. Experiments were carried out at 25 °C.

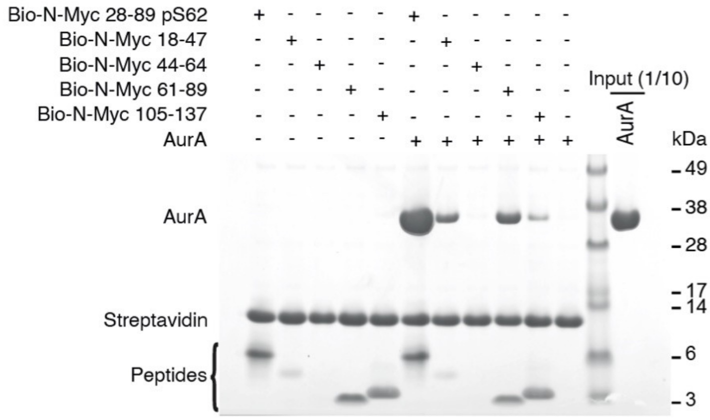

Figure S6. Coprecipitation experiments between immobilised biotinylated N-myc peptides and Aurora-A kinase domain (AurA).

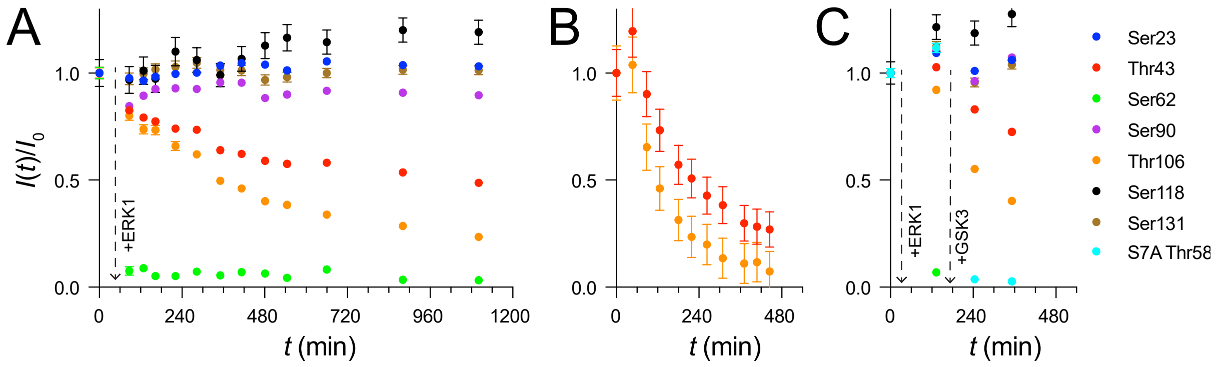

Figure S7. (A) Tracking the kinetics of different sites of phosphorylation by ERK1 within N-myc TAD. Rapid and complete phosphorylation of Ser62 is achieved; much slower phosphorylation of Thr43 and Thr106 is also observed. (B) The rates of off-target ERK1 phosphorylation of Thr43 and Thr106 are increased at higher temperatures (20 °C), though they remain much lower than the rate of Ser62 phosphorylation. (C) Tracking the kinetics of different sites of phosphorylation by ERK1 and GSK3 within N-myc TAD^{S7A}. Rapid and complete phosphorylation of Ser62 by ERK1 is achieved followed by rapid and complete phosphorylation of Thr58; Thr58 does not appear to be significantly affected by ERK1; again, slow phosphorylation of Thr43 and Thr106 (by ERK1) is observed.

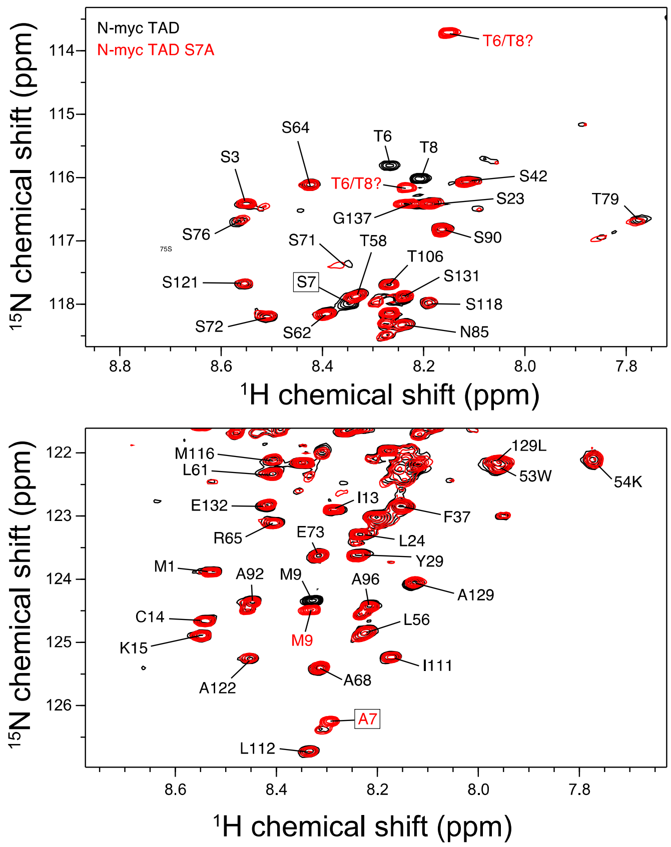

Figure S8. The ^{1}H–^{15}N HSQC spectrum of N-myc TAD^{S7A} (red) shows near-perfect overlap with that of WT N-myc TAD (black). (Upper) the Ser/Thr-rich region of the spectrum. (Lower) the aliphatic-rich region of the spectrum. Differences are the desired loss of the Ser7 peak, gain of a ^{15}N downfield peak attributed to Ala7, a small shift in the peak position of Met9 and unattributed changes to the peak positions for direct neighbours Thr6 and Thr8.

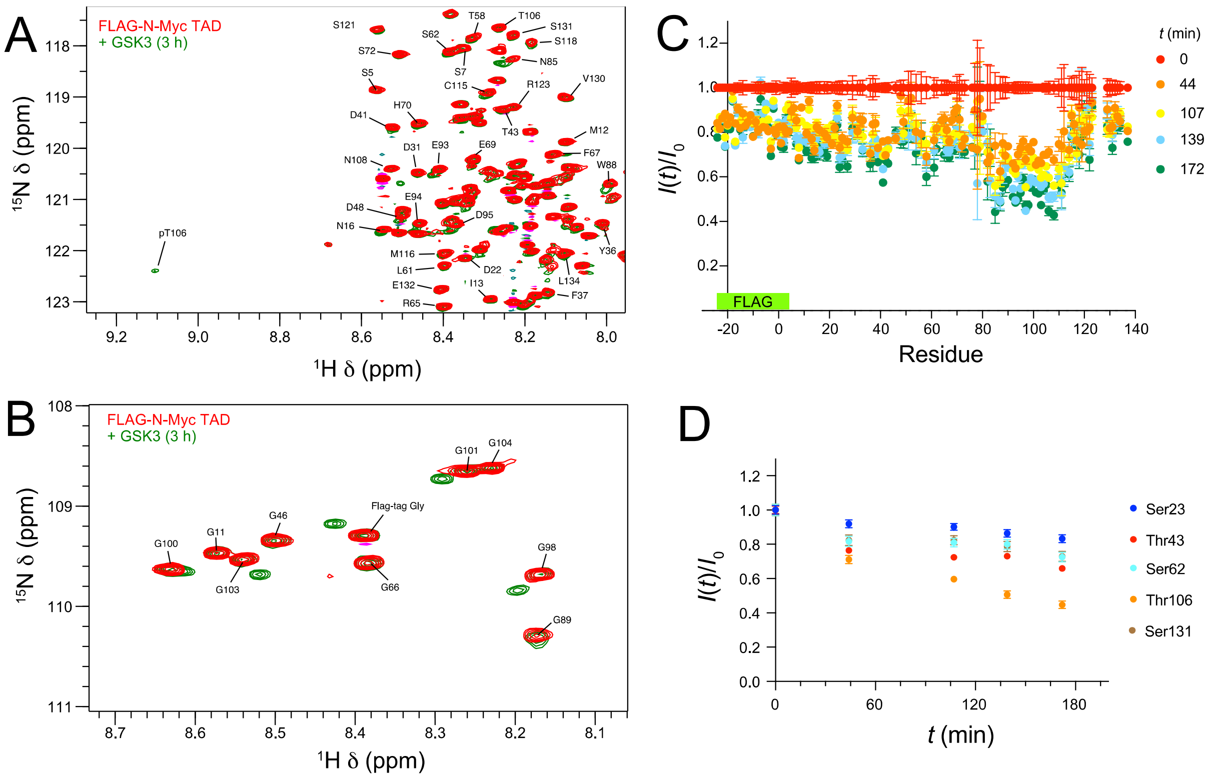

Figure S9. Slow phosphorylation of Thr106 by GSK3 observed using NMR. Treatment of N-myc TAD with GSK3 for 3 h brought about the emergence of a weak downfield phosphothreonine peak (A) as well as five new minor peaks (B) attributed to the nearby Gly-rich region Gly98, Gly100, Gly101, Gly103 and Gly104. (C) Tracking relative peak intensity changes over time after exposure to GSK3 reveals specific peak intensity reductions over a relatively broad range of residues (~85–112), locating the phosphorylation site to Thr106 and suggesting a significant effect of Thr106 phosphorylation on this Gly-rich region. Note: the general loss in peak intensity at the first measured time-point is due to sample dilution on addition of GSK3. (D) Peak intensity ratios for Thr106 and other selected sites as a function of time. The time to reduce Thr106 intensity to 50% was greater than 3 h.

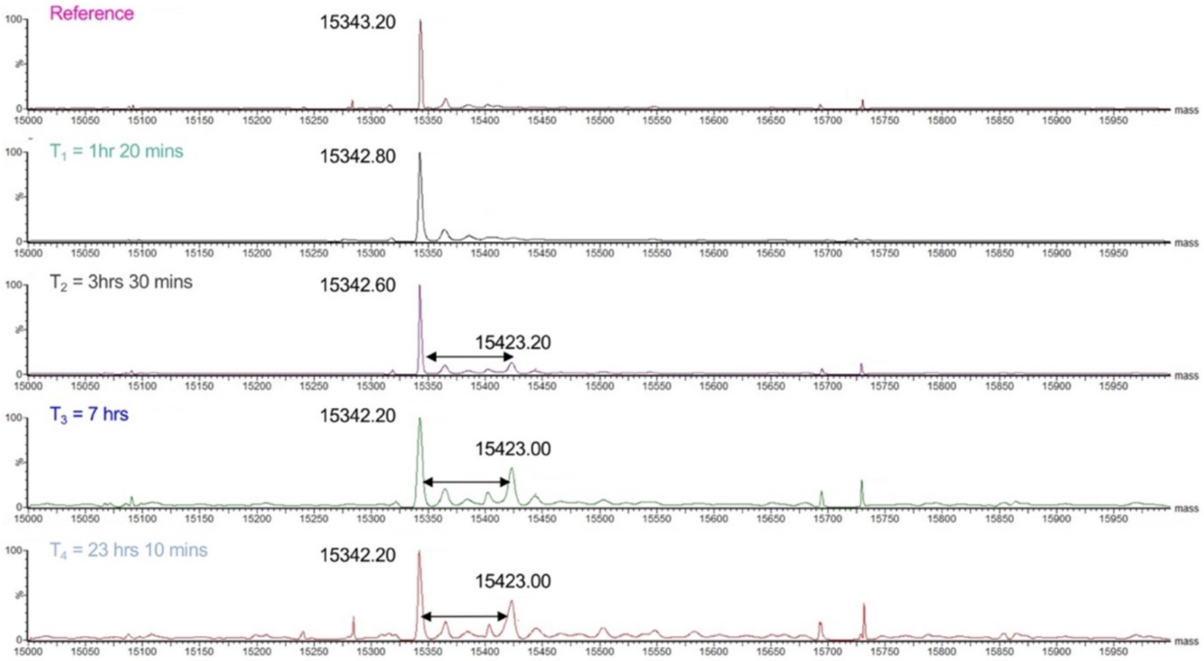

Figure S10. Phosphorylation of Thr58 by GSK3 requires priming phosphorylation at Ser62. Intact mass spectra of N-myc TAD were recorded before and at different timepoints after incubation with GSK3.

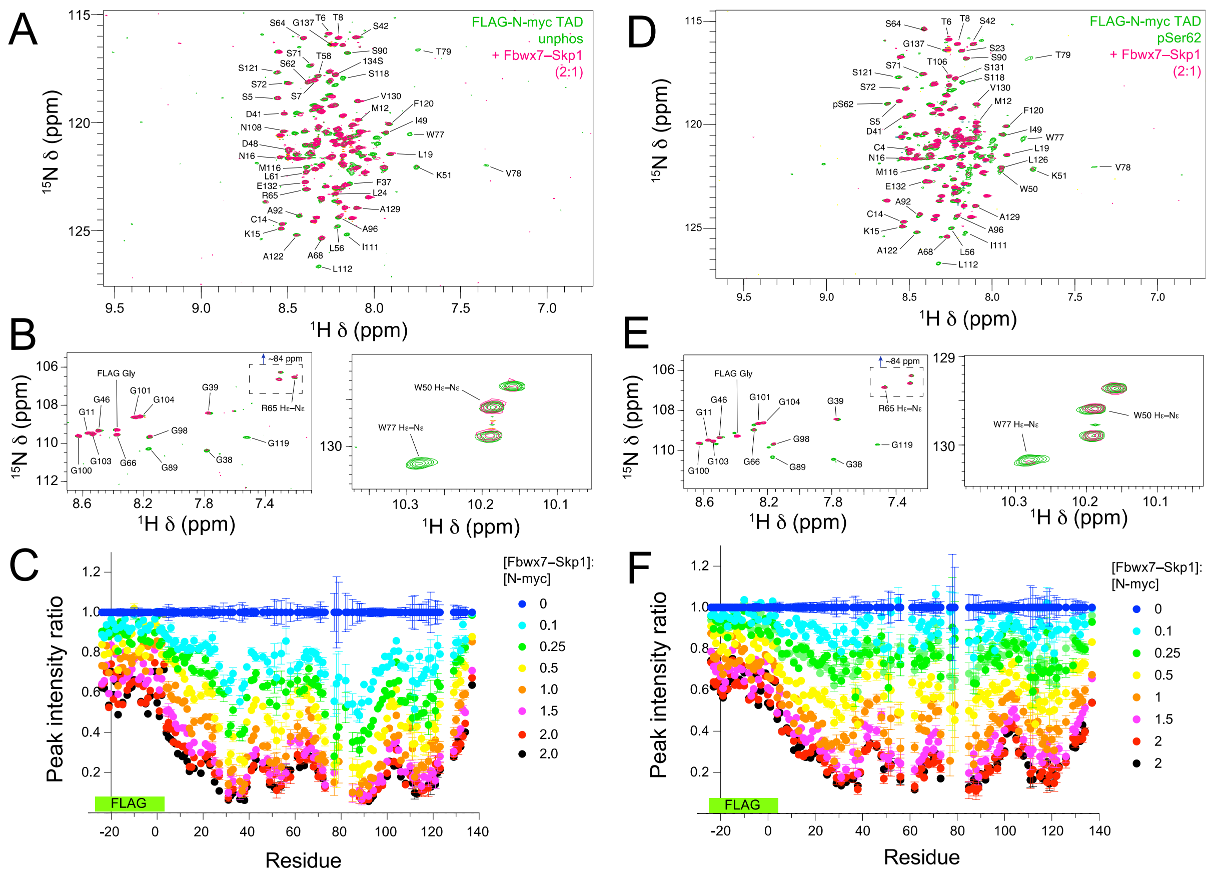

Figure S11. NMR titration of the Fbxw7–Skp1 complex with unphosphorylated (A–C) and *in situ* generated singly phosphorylated N-myc TAD (pSer62) (D–F). (A, D) The central backbone region of the ^{1}H–^{15}N HSQC spectrum before (green) and after addition of Fbxw7–Skp1 (pink). (B, E) Magnified views of the Gly region (left) and Trp Hε–Nε region (right) of the ^{1}H–^{15}N HSQC spectrum. Inset is an aliased region (from 84–85 ppm) showing Arg Hε–Nε correlations. Peaks with the greatest reductions in intensity highlight the parts of the sequence that interact most strongly. (C, F) Ratio of peak intensities for each residue as a function of increasing molar ratio of [Fbxw7–Skp1]:[N-myc].

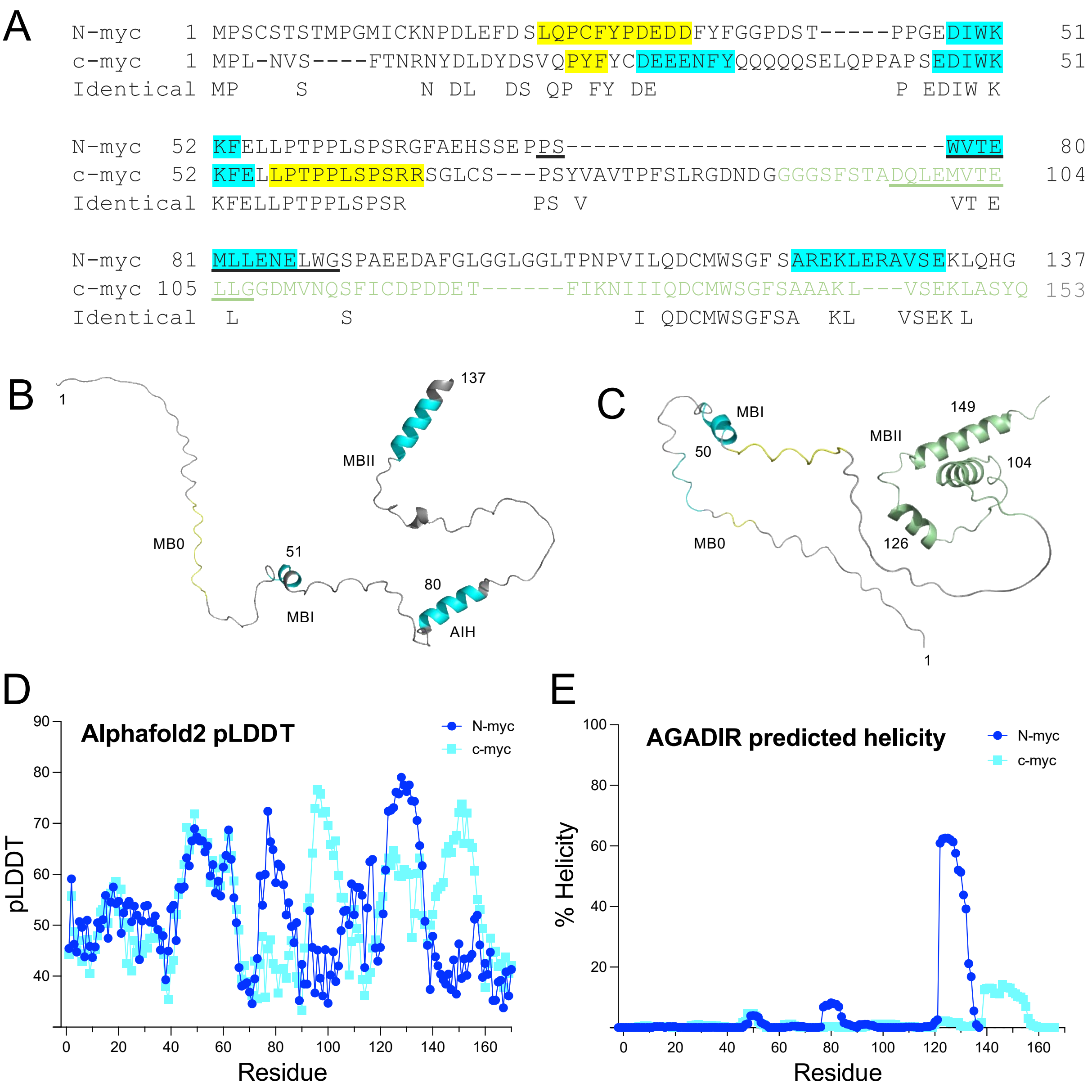

Figure S12. Comparison of secondary structure predictions for N-myc and c-myc. (A) Sequence alignment of TADs from N-myc and c-myc, color-coded to show helical regions (cyan) and beta-turn/strand regions (yellow). The region of c-myc TAD for which structural NMR data are unavailable is shown in light green. Underlined sequences are helices in the crystal structures of these regions of N-myc and c-myc. (B) Structure of N-myc based on Alphafold2 prediction (https://alphafold.ebi.ac.uk/entry/P04198), color-coded as (A). (C) Structure of c-myc based on Alphafold2 prediction (https://alphafold.ebi.ac.uk/entry/P01106), color-coded as (A). (D) Residue-specific pLDDT values from Alphafold2 structure predictions for N-myc and c-myc. An α-helix in N-myc is predicted from 76–88 but with 'low' confidence (average pLDDT score 58.2), whereas the MBII+ helix in N-myc (122–137) is predicted with high confidence (average pLDDT score 71.2).

Likewise the c-myc AlphaFold2 model predicts a helix at the same 'MBII+' position as in N-myc (c-myc 140–155), albeit with reduced confidence (average pLDDT score 64.0). The region of c-myc that binds the TBP–TAF1 complex (Wei *et al.*, [30]) also appears as a helix (average pLDDT score 66.1 for residues 95–107). (E) Output from AGADIR prediction of helical propensity for N-myc and c-myc (http://agadir.crg.es).
